# The changing epidemiology of infection-associated mortality in Alzheimer’s disease in the United States, 1999–2024

**DOI:** 10.3389/fnagi.2026.1885030

**Published:** 2026-07-30

**Authors:** Xuemeng Miao, Yingzhi Zhang, Chengtian Gao, Wenjun Meng, Jinhua Yan, Tao Zeng, Guofang Tang

**Affiliations:** 1 School of Medicine, Xiamen University, Xiamen, Fujian, China; 2 Fujian Provincial Key Laboratory of Neurodegenerative Disease and Aging Research, School of Medicine, Institute of Neuroscience, Xiamen University, Xiamen, Fujian, China; 3 Department of Pain Management, West China Hospital, Sichuan University, Chengdu, China; 4 Department of Comprehensive Special Medical Services, International Medical Center, Sichuan Clinical Research Center for Cancer, Sichuan Cancer Hospital and Institute, Sichuan Cancer Center, University of Electronic Science and Technology of China, Chengdu, China; 5 Department of Medical Oncology, Sichuan Clinical Research Center for Cancer, Sichuan Cancer Hospital and Institute, Sichuan Cancer Center, University of Electronic Science and Technology of China, Chengdu, China; 6 Guangzhou Key Laboratory of Clinical Pathogen Research for Infectious Diseases, Institute of Infectious Diseases, Guangzhou Eighth People’s Hospital, Guangzhou Medical University, Guangdong, Guangzhou, China

**Keywords:** Alzheimer’s disease, epidemiological disparities, infection-associated mortality, multiple cause of death, temporal trends

## Abstract

**Background:**

Infections are major immediate causes of death in patients with Alzheimer’s disease, trends across infection subtypes remain poorly characterized. We examined temporal trends, demographic geographic disparities, COVID-19-era patterns, projected infection-associated mortality in United States, 1999–2024.

**Methods:**

Using CDC Multiple Cause of Death database, infection-associated AD deaths were classified into six subtypes. AAMRs and temporal trends were estimated using joinpoint regression. Kitagawa decomposition assessed demographic age-specific contributions to sex disparities, Bayesian Structural Time Series models projected burden through 2034.

**Results:**

Among 2,342,153 AD-related deaths, 308,045 (13.2%) were infection-associated. Overall infection-associated AAMR declined by 57.6% (AAPC −3.72% per year), diverging from rising AD mortality. Respiratory infections showed the steepest decline (AAMR −68.3%) with improvements across all regions and urbanization strata. By contrast, genitourinary infections were the only subtype with rising burden—absolute deaths increased 75.1%, rural AAMR rose 73.1% over 1999–2020, and central trend-continuation projections suggest a possible further increase, particularly over the near-term horizon, although uncertainty becomes substantial in the longer term. Kitagawa decomposition showed that the female excess in genitourinary mortality was attributable to higher age-specific female rates rather than to population age structure—uniquely among the subtypes examined (cumulative risk effect +11,337; 47.4% of the female excess; cumulative female deaths exceeding male by 170%); the increase in this risk component over time was directionally consistent but of model-dependent statistical significance. Mortality was overwhelmingly concentrated in the oldest age stratum ( ≥ 85-year rate 2,516-fold that of < 65 years). During the COVID-19 era, overall infection-associated AD mortality fell below pre-pandemic projections, with genitourinary infections showing the smallest cumulative deficit of any subtype. Hispanic individuals had the highest AAMR by 2024, while Non-Hispanic Black individuals maintained elevated septicemia mortality throughout.

**Conclusion:**

Although infection-associated AD mortality declined substantially, a growing genitourinary infection burden widened across sex, rural, and racial strata, with trend-continuation projections suggesting a possible further increase, particularly over the near-term horizon. These findings, combined with external clinical evidence on infection prevention, suggest that targeted measures—including CAUTI prevention, sex-specific bladder health programs, and rural infection prevention investment—merit priority attention as baby boomers enter peak AD risk.

## Introduction

Alzheimer’s disease (AD) is the most prevalent neurodegenerative disorder and the leading cause of dementia worldwide, accounting for 60–80% of all dementia cases (World Health Organization, 2021). Globally, more than 55 million individuals are currently living with dementia, and this number is projected to nearly triple by 2050 as populations age (GBD 2019 Dementia Forecasting Collaborators, 2022). In the United States alone, approximately 7.2 million adults aged 65 and older are currently living with AD (Alzheimers Dementia, 2026). Critically, deaths attributed to AD in the United States increased by more than 134% between 2000 and 2024, a trajectory starkly divergent from the concurrent declines observed for heart disease, stroke, and HIV—underscoring that AD represents a mounting rather than receding public health emergency (Alzheimers Dementia, 2026). Without effective medical breakthroughs, the number of Americans with AD is projected to reach 13.8 million by 2060, with the total cost of care in the United States anticipated to approach $1 trillion annually by 2050 (Chen et al., 2024). Together, these trends underscore the rapidly expanding clinical, societal, and economic burden of AD.

Although AD is primarily classified as a neurodegenerative disorder, infections represent one of the leading immediate causes of death in affected patients (Manabe et al., 2017; Manabe et al., 2019). Pneumonia is particularly common in advanced disease and has been identified as a major terminal event in institutionalized patients with dementia (Tolppanen et al., 2020). Previous studies have further shown that patients with AD or related dementias experience substantially elevated mortality following respiratory infections, urinary tract infections, and sepsis (Bouza et al., 2019; Janbek et al., 2021; Järvinen et al., 2023). These compounding vulnerabilities are likely driven by multiple converging biological mechanisms. Age-associated immunosenescence impairs both innate and adaptive immune responses, reducing phagocytosis, attenuating T- and B-lymphocyte activation, and weakening pathogen clearance, thereby rendering patients with AD disproportionately susceptible to severe and recurrent infections (Shaw et al., 2013; Theodorakis et al., 2024). In parallel, disease-specific physiological changes—including dysphagia affecting up to 57% of patients with dementia, impaired cough reflex, urinary incontinence, and prolonged immobility—create structural portals for recurrent infection that cannot be fully mitigated by pharmacological intervention alone (Alagiakrishnan et al., 2013). Importantly, the relationship is bidirectional: systemic infections can further accelerate AD progression through microglial activation, cytokine release, and pathogen-associated molecular pattern-mediated promotion of amyloid-β aggregation, thereby establishing a self-reinforcing cycle between immune vulnerability and neurodegeneration (Ganz et al., 2022; Holmes et al., 2009).

Despite the substantial burden of infection-associated mortality in AD, existing epidemiological evidence remains fragmented. Most prior studies have focused predominantly on pneumonia-related mortality without systematically evaluating the broader spectrum of infectious complications in AD, including genitourinary infections, septicemia/sepsis, digestive infections, and opportunistic infections (Faheem et al., 2026; Sarmad Javaid et al., 2025). In addition, many previous investigations were limited to pre-pandemic or early-pandemic periods, restricting assessment of how the COVID-19 era may have reshaped infection-associated mortality patterns in AD. Furthermore, reliance on underlying-cause-of-death classifications may underestimate the true infectious burden in AD, as infections are frequently recorded as contributing rather than primary causes of death.

To address these gaps, we conducted a comprehensive nationwide population-based analysis of infection-associated AD mortality in the United States from 1999 to 2024 using a multiple-cause-of-death framework. We characterized long-term temporal trends across major infection subtypes, evaluated demographic and geographic disparities, quantified excess mortality during the COVID-19 era, and projected the future burden of infection-associated AD deaths through 2034. Together, these analyses provide a comprehensive overview of the evolving epidemiology of infection-associated mortality in AD across the pre-pandemic, pandemic, and post-pandemic periods.

## Materials and methods

### Data source and case identification

All mortality data were obtained from the Multiple Cause of Death (MCD) database, publicly available through the CDC Wide-ranging Online Data for Epidemiologic Research (CDC WONDER) platform,^1^ covering the period 1999 through 2024 (Friede et al., 1993). The MCD database compiles death certificate records from all 50 US states and the District of Columbia, with cause-of-death information coded according to the International Classification of Diseases, Tenth Revision (ICD-10) (World Health Organization, 2016).

Alzheimer’s disease–related deaths were identified using ICD-10 code G30 (Alzheimer’s disease) recorded as the underlying cause of death. Among these, infection-associated AD deaths were defined as deaths in which at least one infection-related ICD-10 code was additionally documented in any of the multiple-cause (contributing) fields of the same death certificate. This operationalisation—AD as the underlying cause with infection recorded as a multiple (contributing) cause—was adopted because the CDC WONDER platform permits a single underlying cause per record and supports any-mention selection only through the multiple-cause field. It captures infection as a contributor to AD-related death while preserving a consistent denominator (all AD-underlying deaths) for the proportion classified as infection-associated. For analytical purposes, two complementary operational definitions were used to address the asymmetry inherent in death certificate coding: the primary definition (P-definition) identified Alzheimer’s disease (ICD-10 G30) as the underlying cause of death with infection recorded in any contributing cause field, whereas the reciprocal definition (N-definition) identified infection-related ICD-10 codes as the underlying cause with Alzheimer’s disease listed as a contributing cause. Unless otherwise specified, all primary analyses were conducted using the P-definition.

Because a single certificate may list more than one infection site among the contributing causes, a given death may contribute to multiple infection subtypes; subtype proportions therefore sum to more than 100% of unique infection-associated deaths. It should be noted that CDC WONDER delivers mortality data in aggregated, tabulated form—providing counts and rates stratified by user-specified groupings—and does not release individual-level death records through its public interface (Friede et al., 1993). Consequently, it is not possible to directly enumerate the frequency with which multiple infection subtypes co-occur on the same death certificate using this platform. Individual-level mortality microdata with complete multiple-cause fields are available only through the NCHS Research Data Center under a restricted-use data agreement. This structural feature of the database is a recognized limitation shared by all population-based mortality analyses conducted via CDC WONDER (Minhas et al., 2025), and the subtype proportions reported here—which necessarily sum to more than 100% of unique infection-associated deaths when considered collectively—should be interpreted accordingly. Throughout, this outcome is referred to as infection-associated AD mortality as recorded on death certificates: it reflects the documented co-occurrence of AD and infection-related ICD-10 codes on the same certificate and does not constitute a clinically adjudicated determination that infection was the cause of death.

Infection subtypes were classified into six anatomically based categories—infections, genitourinary infections, septicemia/sepsis, digestive system infections, opportunistic infections, and other infections (encompassing cardiovascular, central nervous system, bone, and soft tissue infections)—according to the ICD-10 codes listed in the cause-of-death fields. The full list of ICD-10 codes assigned to each category is provided in Supplementary Tables S1, S2. Several methodological considerations informed this classification scheme. First, because individual death certificates may document more than one infection site, a single death may contribute to multiple infection subtype categories simultaneously; consequently, subtype proportions sum to more than 100% of total unique infection-associated deaths when considered collectively. Second, the septicemia/sepsis category encompasses both explicit sepsis diagnoses and ICD-10 codes designating bacterial or viral agents associated with septic conditions. Third, the “other infections” category consolidates cardiovascular, central nervous system, bone, and soft tissue infections into a single group on the basis of their shared characteristics as deep-seated, invasive infections with individually low case counts.

### Statistical analysis

#### Age-adjusted mortality rates

Age-adjusted mortality rates (AAMRs) were calculated using the direct standardization method, with the year 2000 United States Standard Population as the reference, and are expressed per 100,000 population throughout. AAMRs and their standard errors were extracted directly from CDC WONDER. Approximate 95% confidence intervals for AAMRs were computed as AAMR ± 1.96 × SE (AAMR). Crude mortality rates are additionally reported for age-stratified analyses, where age adjustment is not applicable, and are expressed per 100,000 stratum-specific population. Age-adjusted rates used CDC WONDER bridged-race population denominators. Deaths with counts fewer than 10 in any stratum were suppressed by CDC WONDER in accordance with data use restrictions to protect patient confidentiality; suppressed values were imputed as five (the midpoint of the range 1–9) for count-based quantities, consistent with established practice in population-based mortality research. Separately, CDC WONDER flags rates based on fewer than 20 deaths in the numerator as “unreliable” owing to the large relative standard error; unlike suppressed cells, unreliable cells have both counts and rates published but are flagged as potentially unstable. In the primary analyses, unreliable rates were retained for descriptive reporting but were excluded from joinpoint trend estimation to avoid undue influence of statistically unstable observations. Because CDC WONDER publishes no rate for suppressed cells, the age-standardized rate and joinpoint analyses excluded both suppressed and unreliable cells (complete-case) and are therefore arithmetically independent of the chosen imputation value; however, the exclusion of these cells means that some annual observations are absent from the rate-based trend estimation for the lowest-count strata, constituting a data-availability limitation even though the surviving rates are unaffected by the imputation. The scope and robustness of the imputation are examined in Sensitivity analyses (Supplementary Table S3).

#### Subgroup analyses

Temporal trends in AAMR were examined across five stratification dimensions: sex (female, male); age group ( < 65, 65–74, 75–84, and ≥ 85 years), with crude mortality rates reported for each stratum given the inapplicability of age adjustment within age-specific analyses; race/ethnicity (Non-Hispanic White, Non-Hispanic Black, Hispanic, and Non-Hispanic Asian/Pacific Islander), consistent with CDC WONDER reporting categories; urbanization level (urban, suburban, and rural), classified using the 2013 NCHS Urban–Rural Classification Scheme for Counties, which is available in CDC WONDER only through 2020—urbanization-stratified analyses therefore span 1999–2020, whereas all other analyses span the full 1999–2024 study period; and geography, examined at both the US Census region level (Northeast, Midwest, South, West) and the individual state level. To further characterize sex disparities, the male-to-female AAMR ratio was calculated annually for each infection subtype and its temporal trend assessed by ordinary least squares (OLS) regression on calendar year. Trends in the annual absolute sex difference in death counts (female minus male) were examined by OLS regression, with statistical significance defined at α = 0.05.

#### Kitagawa decomposition

To disentangle whether observed sex differences in infection-associated AD mortality counts reflected differences in age-specific mortality rates versus differences in population age structure, we applied the Kitagawa decomposition method annually for each infection subtype (Cheng et al., 2019). This approach partitions the absolute difference in death counts between females and males into two additive components: a population size effect (attributable to differences in the number and age distribution of the at-risk population between sexes) and a risk effect (attributable to differences in age-specific mortality rates between sexes, independent of population structure). A positive risk effect indicates higher age-specific mortality rates in females, net across age strata; a negative value indicates the converse. This decomposition is arithmetic—it apportions the death-count difference between a rate component and a population-structure component—and does not by itself identify any biological, clinical, or individual-level mechanism, nor distinguish between-sex differences in exposures such as catheterisation, institutionalization, healthcare use, diagnosis, or death-certificate coding. Cumulative components over 1999–2024 were obtained by summing annual values, and temporal trends in each component were assessed by ordinary least squares regression on calendar year (two-sided α = 0.05).

#### Joinpoint regression analysis

Temporal trends in AAMRs were examined using joinpoint regression analysis, implemented in R using the segmented package (Muggeo, 2003). This segmented log-linear modeling approach identifies statistically significant inflection points at which the direction or magnitude of a mortality trend changes, and estimates the Annual Percent Change (APC) within each resulting time segment. The optimal number of joinpoints (maximum: 5) was selected using the Bayesian Information Criterion. The Average Annual Percent Change (AAPC)—a summary measure of the overall trend across the full study period (1999–2024)—was calculated as a segment-length weighted average of the segment-specific APCs. Segmented AAPCs were additionally computed for the pre-COVID (1999–2019) and post-COVID (2020–2024) sub-periods. All estimates are reported with 95% confidence intervals; a two-sided *p*-value < 0.05 was considered statistically significant.

#### Mortality projections (2025–2034)

Future mortality burden was projected using Bayesian Structural Time Series (BSTS) models with a local linear trend specification, implemented in R using the bsts package (Scott and Varian, 2014). To account for the pronounced age gradient in infection-associated AD mortality and the differential demographic shifts across age strata, projections were performed at the age-specific rate level rather than on aggregate mortality rates. Analyses were restricted to individuals aged 60 years and older, stratified into 6, 5-year age groups (60–64, 65–69, 70–74, 75–79, 80–84, and ≥ 85 years). Because CDC WONDER does not provide 5-year age group population denominators for the ≥ 85-year stratum, the corresponding population counts were obtained from the 10-year age group file (aggregating the 85–89, 90–94, 95–99, and ≥ 100-year strata) and used as the denominator for that group throughout.

For each infection subtype and age group, a BSTS local linear trend model was fitted to the log-transformed age-specific mortality rate series (1999–2024). This specification allows both the level and slope of the trend to evolve over time, providing flexibility to capture non-linear trajectories. Predicted rates for 2025–2034 were obtained from the posterior predictive distribution (3,000 MCMC iterations; 500 burn-in), with point estimates and 95% prediction intervals (PIs) derived from the posterior mean and the 2.5th–97.5th percentiles, respectively. Projected annual death counts were calculated by multiplying predicted age-specific rates by the corresponding US Census Bureau national population projections (2014–2060 series), summed across six age groups (60–64 through ≥ 85 years) to obtain subtype-level totals. Prediction intervals reflect rate uncertainty only and do not incorporate population projection uncertainty; true uncertainty in projected death counts is therefore wider than reported.

#### Model validation and sensitivity analysis

To select the primary projection method, a holdout validation was conducted (training: 1999–2018; validation: 2019–2024), comparing three candidate approaches: BSTS local linear trend, ARIMA (order auto-selected by AIC via the forecast package), and log-linear extrapolation (Hyndman and Khandakar, 2008). Model performance was evaluated using MAPE, RMSE, mean bias, and 95% PI coverage. BSTS was selected on the basis of superior PI calibration (near-nominal 95% coverage versus 50–75% for ARIMA and linear extrapolation), although wide prediction intervals contributed to high coverage and high coverage should not be equated with strong point-prediction performance. All three models exhibited systematic positive forecast bias during the holdout period, likely reflecting in part pandemic-era structural changes but constituting a limitation of out-of-sample point prediction regardless of the underlying cause. Sensitivity analyses applied optimistic (−0.5 percentage points/year) and pessimistic (+0.5 percentage points/year) shifts to the baseline trend slope. To characterize the precision of the forward projections, we additionally quantified the relative width of the 95% prediction interval—defined as (upper − lower)/point estimate—for each subtype across the 10-year horizon, and summarized the direction and calibration of the holdout errors by subtype (Supplementary Table S4).

#### COVID-era excess mortality analysis

To contextualize the post-2020 mortality decline, excess deaths during 2020–2024 were estimated by comparing observed deaths against BSTS-projected expected deaths based on the pre-pandemic trend (training: 1999–2019), fitted to log-transformed annual death counts (ages ≥ 60) (Beaney et al., 2020; Leon et al., 2020). Excess deaths were defined as observed minus expected; negative values indicate a net protective effect relative to trend. Cumulative excess deaths and percentage deviations were calculated for each infection subtype. Prediction intervals reflect BSTS trend uncertainty only and do not incorporate uncertainty from population size, ICD-10 coding changes, or model specification. Projection models were restricted to ages ≥ 60, consistent with Census Bureau projection strata; the 2024 baseline (*n* = 7,837) therefore excludes 21 deaths occurring below age 60. Minor discrepancies between tabulated death counts and Kitagawa-decomposed totals reflect cumulative rounding across age strata and study years. To formally assess statistical significance, observed annual deaths were additionally compared against the 95% prediction interval of the pre-pandemic model; a deviation was considered statistically significant only if the observed value fell outside this interval (Supplementary Table S5).

### Sensitivity analyses

#### Cause-of-death coding position

**FIGURE 1:**
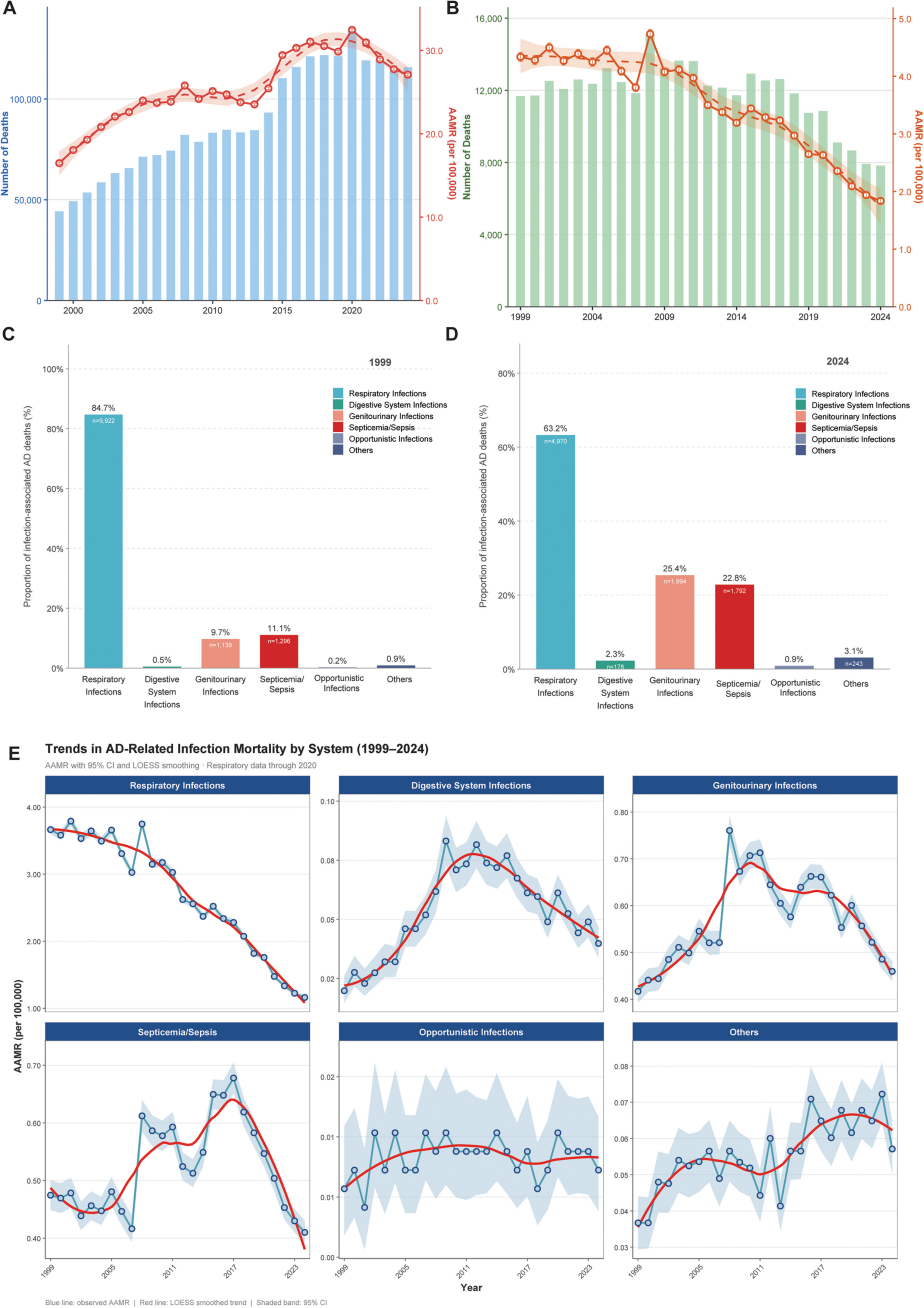
Trends in Alzheimer’s disease mortality and infection-associated subtypes in the United States, 1999–2024. **(A)** Annual number of AD-related deaths and age-adjusted mortality rate (AAMR) from 1999 to 2024. **(B)** Annual number of infection-associated AD deaths and corresponding AAMR over the same period. **(C,D)** Bar charts illustrating the proportional contribution of six infection subtypes to total infection-associated AD deaths in 1999 (C) and 2024 **(D)**. Proportions are calculated as the number of deaths with each infection subtype recorded on the death certificate divided by the total number of unique infection-associated AD deaths in that year (1999: *N* = 11,715; 2024: *N* = 7,858), multiplied by 100. Values sum to more than 100% within each year because individual death certificates may simultaneously list multiple infection subtypes under the multiple-cause-of-death framework; this is a methodological feature of the CDC WONDER platform, not an error. **(E)** AAMR trends for six infection subtypes from 1999 to 2024. AAMR, age-adjusted mortality rate; CI, confidence interval; “Other infections”, cardiovascular, central nervous system, bone, and soft tissue infections.

To assess robustness to cause-of-death coding position, we repeated the analysis under a reciprocal case definition (N-definition), taking infection-related codes as the underlying cause and AD (G30) as any contributing cause. Because each certificate carries a single underlying cause, the N-definition assigns each death to one infection category, making subtypes mutually exclusive (proportions summing to 100%) and directly addressing the > 100% totals inherent to the multiple-cause framework in Figures 1C,D; the P and N definitions are the only two operationalisations estimable from the CDC WONDER aggregate interface. Two documented changes to NCHS ICD-10/ACME coding conventions taking effect with the 2008 data year (see Results) each abruptly reduced the underlying-cause (N-definition) share of one infection subtype—respiratory and genitourinary, respectively—producing abrupt single-year discontinuities in the N-definition series for these two subtypes; separately, the underlying-cause (N/P) share declined progressively across all six subtypes over the study period. Full-period N-definition AAPCs are correspondingly distorted and are reported descriptively only. Directional concordance was therefore assessed in the post-2008 window (2009–2024), in which the two definitions are more directly comparable, by agreement of AAPC sign, overlap of 95% CIs, and the Spearman rank correlation of annual AAMR trajectories. Genitourinary infection—suppressed under the N-definition from 2008 and reportable only for 1999–2007—was not assessable under the N-definition.

#### Scope and impact of suppressed-count imputation

CDC WONDER suppresses counts below 10 without publishing a corresponding rate. Age-standardized rates therefore cannot be reconstructed for suppressed cells, so the AAMR joinpoint analyses excluded these cells (complete-case) and are independent of the imputed value; imputation of suppressed counts to five entered only the count-based summaries (absolute and cumulative tallies and the Kitagawa decomposition). To characterize suppression, we (i) tabulated the proportion of analytical cells (stratum × subtype × year) suppressed in the race/ethnicity (1999–2024) and urbanization (1999–2020) analyses, and (ii) in the subgroups most exposed to suppression, re-estimated trends with crude-rate joinpoint models under four assumptions for suppressed counts (1, 5, 9, or complete-case), reporting the age-standardized complete-case estimate as reference. Crude rates were used only to gage directional stability and did not replace the age-standardized estimates (Supplementary Table S3).

#### Autocorrelation-robust trend inference

Because annual series may exhibit serial autocorrelation that deflates OLS standard errors, all trends in the Kitagawa components—and, for completeness, in the annual female-minus-male death-count differences—were re-estimated with autocorrelation-robust inference. OLS slopes were retained as point estimates, with *p*-values recomputed using Newey–West heteroskedasticity- and autocorrelation-consistent (HAC) standard errors (automatic bandwidth) and AR(1)-GLS as a model-based cross-check; residual autocorrelation was assessed by the Durbin–Watson statistic. For series in which the autoregressive parameter approached the non-stationary boundary (φ→ 1)—a regime in which the AR(1)-GLS transformation approximates first-differencing and, at 26 annual observations, cannot be reliably distinguished from a trend-stationary series with strong but stationary autocorrelation—neither specification was treated as authoritative; trends for which the two specifications disagreed are reported as significant under HAC but attenuated under AR(1)-GLS and are described as of model-dependent significance rather than as established (Supplementary Table S7).

#### Formal between-group comparison of age-adjusted rates

To test whether between-group differences in age-adjusted infection-associated AD mortality were statistically significant—in particular, whether Hispanic patients had a significantly higher AAMR than other racial/ethnic groups in the most recent year—AAMRs were compared using a two-sample z-test for the difference of two independent age-adjusted rates. Because the racial/ethnic populations are mutually exclusive, the corresponding AAMR estimates are independent. Standard errors were derived from the published CDC WONDER 95% confidence intervals as SE = (upper − lower)/(2 × 1.96); the standard error of the difference as SE (D) = √(SE${}_{1}^{2}$ + SE${}_{2}^{2}$); and the test statistic as z = D/SE (D), with a two-sided *p*-value of 2Φ(−|z|) and a 95% confidence interval for the difference of D ± 1.96 × SE (D). The test was applied to all pairwise comparisons among the four racial/ethnic groups for all infections combined, and, for each infection subtype, to the comparison between the highest- and second-highest-ranked groups and between Hispanic and Non-Hispanic White patients, all in 2024 (the most recent year with reportable rates for all groups). This procedure assumes approximately symmetric confidence intervals, which holds for the large counts underlying the all-infection rates; for the lowest-count subtypes the gamma-based CDC WONDER intervals are mildly asymmetric and the corresponding tests are interpreted more cautiously. These comparisons are exploratory; no adjustment for multiplicity was applied, and the *p*-values should be interpreted as descriptive measures of the strength of between-group separation rather than as confirmatory hypothesis tests.

### Complementary mixed-effects count model

As a complement to the joinpoint regression, we fitted a mixed-effects negative binomial model to infection-associated AD death counts, with the logarithm of the population as an offset, to test formally for interactions between calendar year, infection subtype, and sex. To preserve exact counts and denominators without imputation, the model was restricted to the unsuppressed elderly strata (65–74, 75–84, and ≥ 85 years; 624 stratum-year observations, 1999–2024). Calendar year was centered and entered continuously; fixed effects comprised year, infection series, sex, and age group with the year × series, year × sex, and series × sex interactions; and a random intercept for demographic stratum (series × sex × age group) accounted for repeated annual observations. The negative binomial specification was selected over Poisson on the basis of overdispersion, and model adequacy was confirmed with simulation-based residual diagnostics.

### Software

All statistical analyses were performed in R (version 4.2.3), using the segmented package for joinpoint regression, bsts for Bayesian structural time series modeling, and forecast for ARIMA model fitting. A two-sided significance threshold of α = 0.05 was applied throughout. As all data are publicly available, de-identified, and aggregated, no ethical approval was required.

## Results

### Trends in AD-related mortality and infection-associated deaths, 1999–2024

Between 1999 and 2024, a total of 2,342,153 AD-related deaths were recorded in the United States, with annual deaths more than doubling (44,536–116,022; AAPC +4.00% per year) and AAMR rising from 16.47 to a pandemic-era peak of 32.45 per 100,000 in 2020 before declining to 27.09 by 2024 (overall +64.4%; AAPC +1.81% per year; Figure 1A and Supplementary Table S8). Joinpoint regression identified five inflection points across the study period, with the steepest rise observed between 1999 and 2003 (APC +7.48%; 95% CI +6.43% to +8.53%) and a significant decline after 2020 (APC −4.61%; 95% CI −5.93% to −3.28%; Supplementary Table S9). The mean AAMR increased from 24.50 per 100,000 in the pre-COVID era (1999–2019) to 29.43 in the post-COVID era (2020–2024; +20.1%; Supplementary Figure S1B).

Over the same period, 308,045 infection-associated AD deaths were recorded (∼13.2% of all AD deaths), declining from 11,715 in 1999 to 7,858 in 2024 (−32.9%), with AAMR falling from 4.34 to 1.84 per 100,000 (−57.6%; AAPC −3.72%; 95% CI −4.39% to −3.05% per year; Figure 1B and Supplementary Table S8)—a trajectory starkly divergent from overall AD mortality. Joinpoint regression identified two inflection points (2008 and 2017): the AAMR was broadly stable through 2008 (APC −0.12%; *p* = 0.87), declined significantly through 2017 (APC −3.94%; *p* < 0.001), and accelerated markedly thereafter (APC −7.89%; *p* < 0.0001), with further acceleration in the post-COVID era (segmented AAPC −8.74% per year; Supplementary Figure S1 and Supplementary Table S9).

The composition of infection subtypes shifted substantially over the study period (Figures 1C,D). Respiratory infections remained predominant, though their proportional contribution fell from 121.2 to 89.8% of total infection-associated deaths, while genitourinary infections (13.9–36.0%) and septicemia/sepsis (15.8–32.4%) grew in relative prominence. At the subtype level, respiratory infections showed the steepest absolute decline (AAMR −68.3%; AAPC −4.90% per year), accelerating from −5.10% per year (2008–2017) to −9.62% per year thereafter. Digestive system infections rose sharply through 2009 (APC +14.60%; *p* < 0.0001) before reversing after 2014 (APC −5.47%; *p* < 0.0001). Genitourinary infections and septicemia/sepsis each followed biphasic patterns with earlier rises and subsequent significant declines post-2016 and post-2017, respectively. Other infections increased progressively throughout (AAPC +1.72%; 95% CI +1.10% to +2.34%; *p* < 0.0001), while opportunistic infections remained stable (AAPC +0.16%; *p* = 0.62). All six subtypes exhibited accelerated declines or marked attenuation of prior increases during 2020–2024 (Figure 1E and Supplementary Table S9).

**FIGURE 2:**
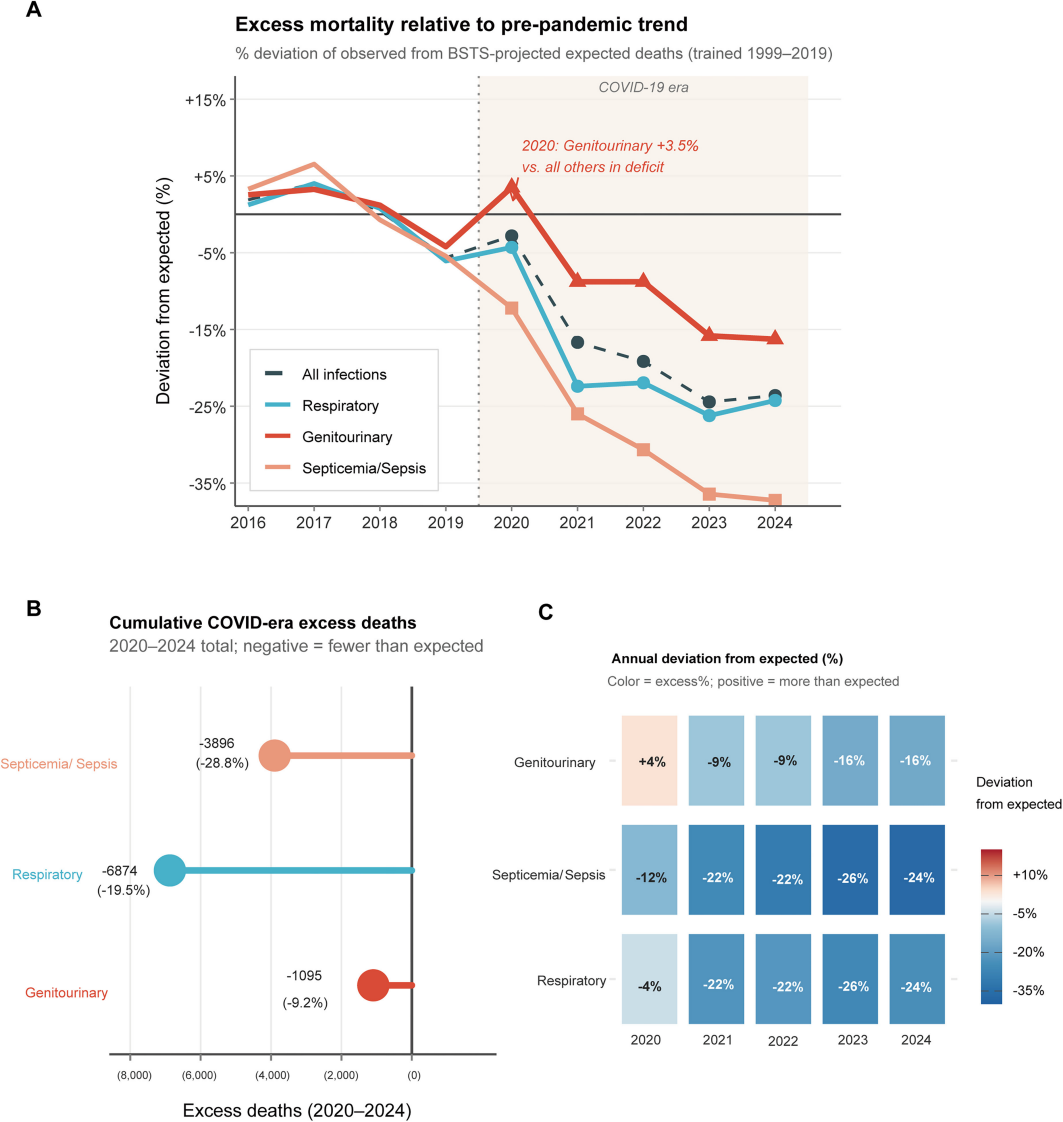
COVID-19 era excess mortality in infection-associated Alzheimer’s disease relative to pre-pandemic projected trajectories, United States, 2020–2024. **(A)** Annual percentage deviation of observed infection-associated AD deaths from BSTS-projected expected deaths for four infection subtypes (all infections combined, respiratory infections, genitourinary infections, and septicemia/sepsis), displayed from 2016 to 2024. **(B)** Cumulative excess deaths over 2020–2024 for three major infection subtypes. **(C)** Heatmap of annual percentage deviation from expected deaths for genitourinary infections, septicemia/sepsis, and respiratory infections across each year of the COVID-19 era (2020–2024). AD, Alzheimer’s disease; BSTS, Bayesian Structural Time Series; COVID-19, coronavirus disease 2019.

During the COVID-19 era (2020–2024), observed infection-associated AD deaths fell 17.1% below pre-pandemic projected levels (observed: 44,343; expected: 53,513), with the deepest cumulative deficits recorded for septicemia/sepsis (−28.8%) and respiratory infections (−19.5%; Figure 2). Genitourinary infections showed by far the smallest cumulative deficit (−9.2%), roughly one-third that of respiratory and septicemia infections, and 2020 was the only subtype-year—across all four subtypes and all 5 years—in which observed deaths exceeded the pre-pandemic point projection (+83 deaths; +3.5%, Figure 2). A formal test against the pre-pandemic 95% prediction interval (Supplementary Table S5), however, showed that this 2020 deviation fell within the interval (expected 2,368; observed 2,451; 95% PI 1,815–3,027) and is therefore not a statistically significant excess; indeed, owing to the width of the BSTS prediction intervals, none of the single-year COVID-era deviations in any subtype reached significance against the 95% PI. The COVID-era comparison is accordingly best interpreted descriptively, in terms of the direction and cumulative magnitude of deviations rather than single-year significance. So interpreted, the relative attenuation of the genitourinary deficit may reflect several non-exclusive mechanisms, including an anatomically driven etiology less amenable to the respiratory-focused non-pharmaceutical interventions that reduced respiratory and septicemia mortality, compromised hygiene-dependent catheter and continence care under pandemic-era staffing shortages, or co-documentation of urinary-tract infection among COVID-19 decedents under the any-mention framework (see Discussion). The aggregate mortality data do not distinguish among these hypotheses. Because pathogen-specific ICD-10 coding was available for only 2.4% of infection-associated AD deaths (7,289 of 308,045), a descriptive, hypothesis-generating characterization of pathogen composition and age-adjusted trends is provided in Supplementary Figures S2, S3 rather than in the main analysis.

### Sex differences in AD-related infection-associated mortality

**FIGURE 3:**
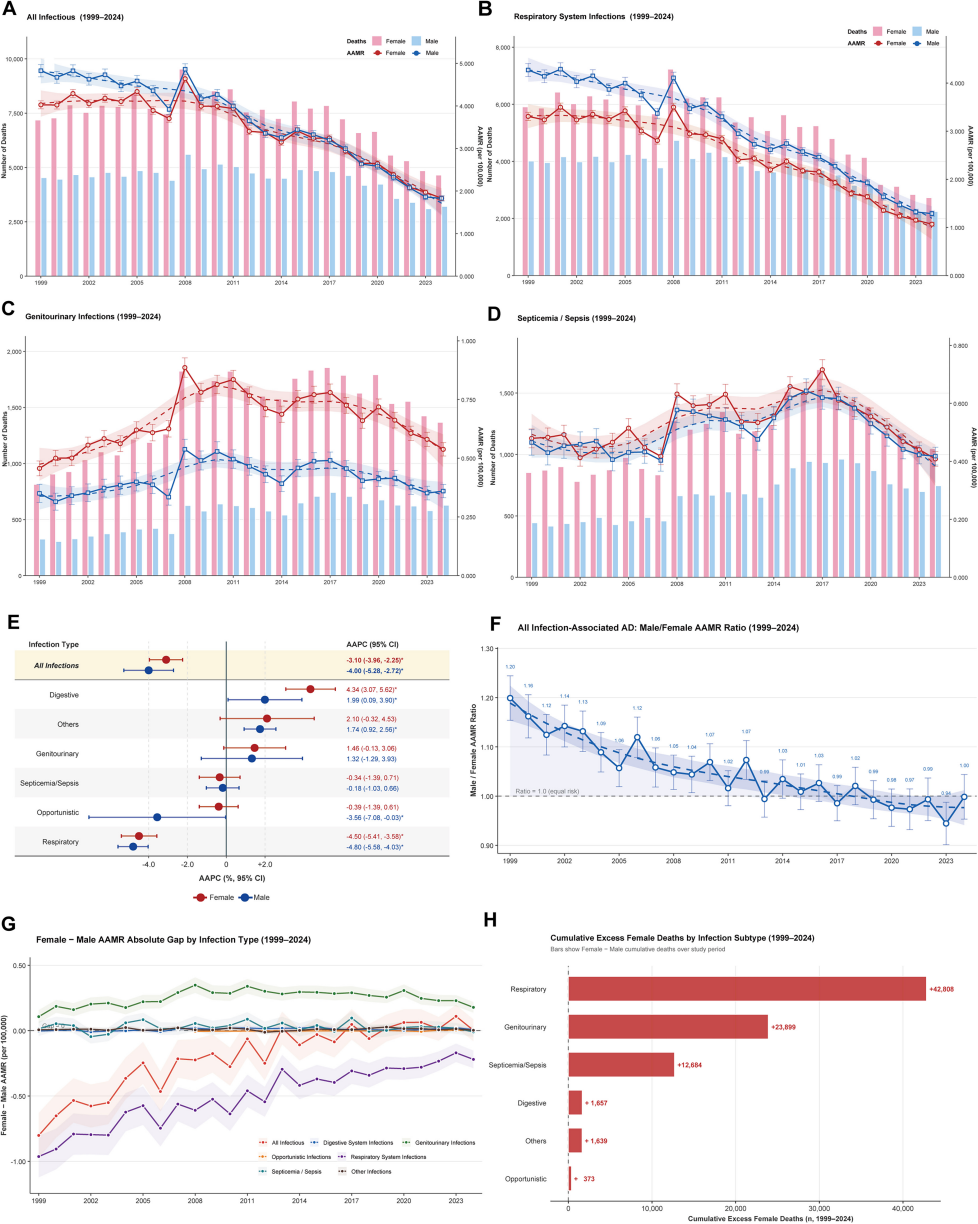
Sex differences in infection-associated Alzheimer’s disease mortality, United States, 1999–2024. **(A–D)** Annual death counts and age-adjusted mortality rates (AAMR) for female (red) and male (blue) Alzheimer’s disease (AD) patients, stratified by infection subtype: **(A)** all infection-associated AD deaths combined, **(B)** respiratory system infections, **(C)** genitourinary infections, and **(D)** septicemia/sepsis. **(E)** Forest plot of average annual percent change (AAPC) in AAMR by infection subtype and sex. **(F)** Temporal trend in the male-to-female AAMR ratio for all infection-associated AD deaths combined (1999–2024). **(G)** Annual absolute AAMR gap by infection subtype, with shaded 95% confidence bands; positive values indicate female predominance and negative values indicate male predominance. **(H)** Cumulative excess female deaths (female minus male death counts, 1999–2024) by infection subtype. AAMR, age-adjusted mortality rate per 100,000 population; AAPC, average annual percent change; CI, confidence interval.

Sex differences in AAMR exhibited markedly heterogeneous patterns across infection subtypes (Figures 3A,E). For all infection-associated AD deaths combined, male AAMR exceeded female AAMR at baseline (4.83 vs. 4.03 per 100,000 in 1999) but converged to near-parity by 2024 (1.83 vs. 1.83), with males declining at a significantly faster rate (AAPC −4.00% vs. −3.10% per year). Respiratory infections—the dominant subtype—showed consistently higher male AAMR throughout despite a larger absolute female death burden (138,097 vs. 95,289), with broadly comparable rates of decline in both sexes (female AAPC −4.50%; male AAPC −4.80%; Figure 3B). By contrast, genitourinary infections demonstrated persistent and widening female predominance: female AAMR rose from 0.457 to 0.538 per 100,000 (+17.8%) while male AAMR remained nearly flat (+2.8%), yielding 37,934 cumulative female deaths versus 14,035 male deaths—a female excess of 23,899 deaths, or 170% above the male total (Figure 3C). Septicemia/sepsis showed near-parity between sexes with non-significant AAPCs in both (Figure 3D), while digestive system infections showed the steepest female divergence in trend (female AAPC +4.34% vs. male +1.99%; Supplementary Figure S4).

The male-to-female AAMR ratio declined progressively from 1.199 in 1999 to 0.998 in 2024 (slope −0.0078/year, *p* < 0.001; Figure 3F), crossing below unity around 2019. This overall convergence was driven principally by the narrowing respiratory infection gap (−64.74 deaths/year, *p* < 0.001). Genitourinary infections diverged in the opposite direction, consistent with the faster rise in female than male genitourinary AAMR noted above; the slope of the annual female-minus-male genitourinary death-count difference (OLS +14.82 deaths/year) was, however, not statistically significant under autocorrelation-robust (HAC) inference (*p* = 0.072) and is not interpreted as a robustly widening annual difference (Figures 3G,H). Cumulatively, females accounted for +74,173 more infection-associated AD deaths than males across the study period, with the largest contributions from respiratory infections (+42,808), genitourinary infections (+23,899), and septicemia/sepsis (+12,684; Figure 3H).

### Age-stratified trends in infection-associated AD mortality

**FIGURE 4:**
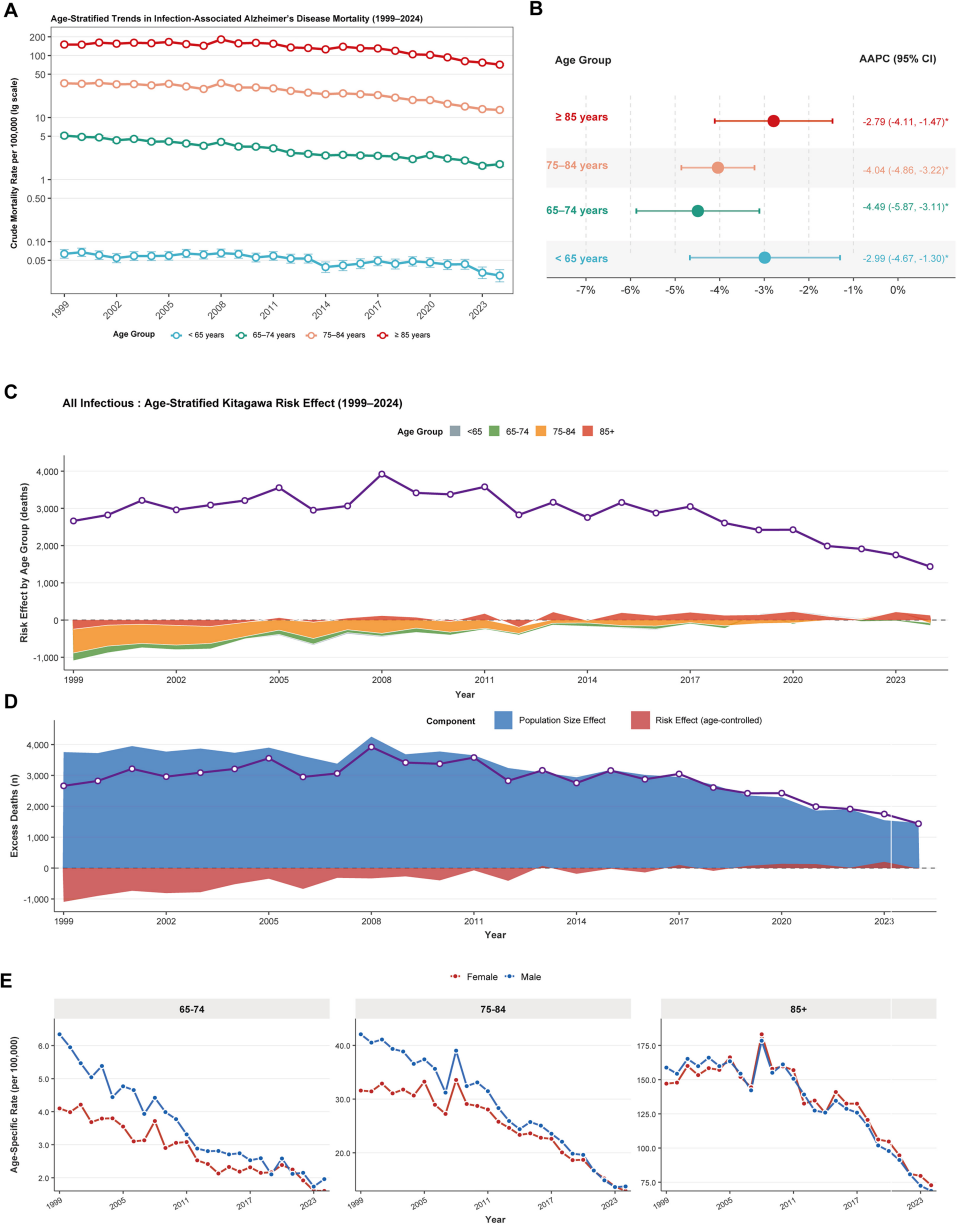
Age-stratified trends and kitagawa decomposition of infection-associated Alzheimer’s disease mortality, United States, 1999–2024. **(A)** Age-stratified crude mortality rates (per 100,000) for infection-associated AD deaths across four age groups ( < 65, 65–74, 75–84, and ≥ 85 years). **(B)** Forest plot of AAPCs in age-specific crude mortality rates by age group **(C)** Age-stratified Kitagawa risk effect (deaths attributable to differences in age-specific mortality rates between sexes, net of population age structure) for all infection-associated AD deaths combined, stratified by age group ( < 65, 65–74, 75–84, ≥ 85 years). **(D)** Annual Kitagawa decomposition of the total observed female excess death burden into two components: population size effect and age-controlled risk effect **(E)** Age-specific mortality rates by sex across three elderly age strata (65–74, 75–84, and ≥ 85 years). AAPC, average annual percent change; AD, Alzheimer’s disease; CI, confidence interval.

Crude mortality rates for infection-associated AD deaths declined significantly across all four age groups over the study period, yet with markedly heterogeneous magnitudes (Figures 4A,B). The steepest relative declines were observed in the 65–74 and 75–84 years groups (AAPC −4.49 and −4.04%, respectively), whereas the youngest ( < 65 years; AAPC −2.99%) and oldest ( ≥ 85 years; AAPC −2.79%) groups showed the slowest rates of improvement—all four AAPCs statistically significant. Despite these declines, the age gradient remained extraordinarily steep throughout the study period: in 2024, the crude mortality rate in the ≥ 85 years group (71.25 per 100,000) exceeded that of the < 65 years group (0.03 per 100,000) by a factor of 2,516, underscoring the overwhelmingly age-concentrated nature of infection-associated AD mortality. Age-specific mortality rates by sex across the three elderly strata (65–74, 75–84, and ≥ 85 years) are shown in Figure 4E.

### Kitagawa decomposition: disentangling risk from demographics

For all infection-associated AD deaths combined, the cumulative female excess of +74,210 was almost entirely attributable to the population size effect (+81,581; 109.9%), while the cumulative risk effect was negative (−7,371; −9.9%), indicating that male AD patients consistently carried higher age-specific infection mortality rates within each age stratum (Figures 4C,D). Notably, the risk effect increased significantly over time (+43.44 deaths/year, *p* < 0.001) while the population size effect declined (−93.77 deaths/year, *p* < 0.001), suggesting a gradual erosion of male predominance in age-specific risk. Respiratory infections represented the most extreme demographic artifact, with a strongly negative cumulative risk effect (−20,899; −48.8%) and the entire apparent female excess (+42,821) attributable to population structure; the risk effect nonetheless trended upward (+31.87/year, *p* < 0.001; Supplementary Figure S5).

**FIGURE 5:**
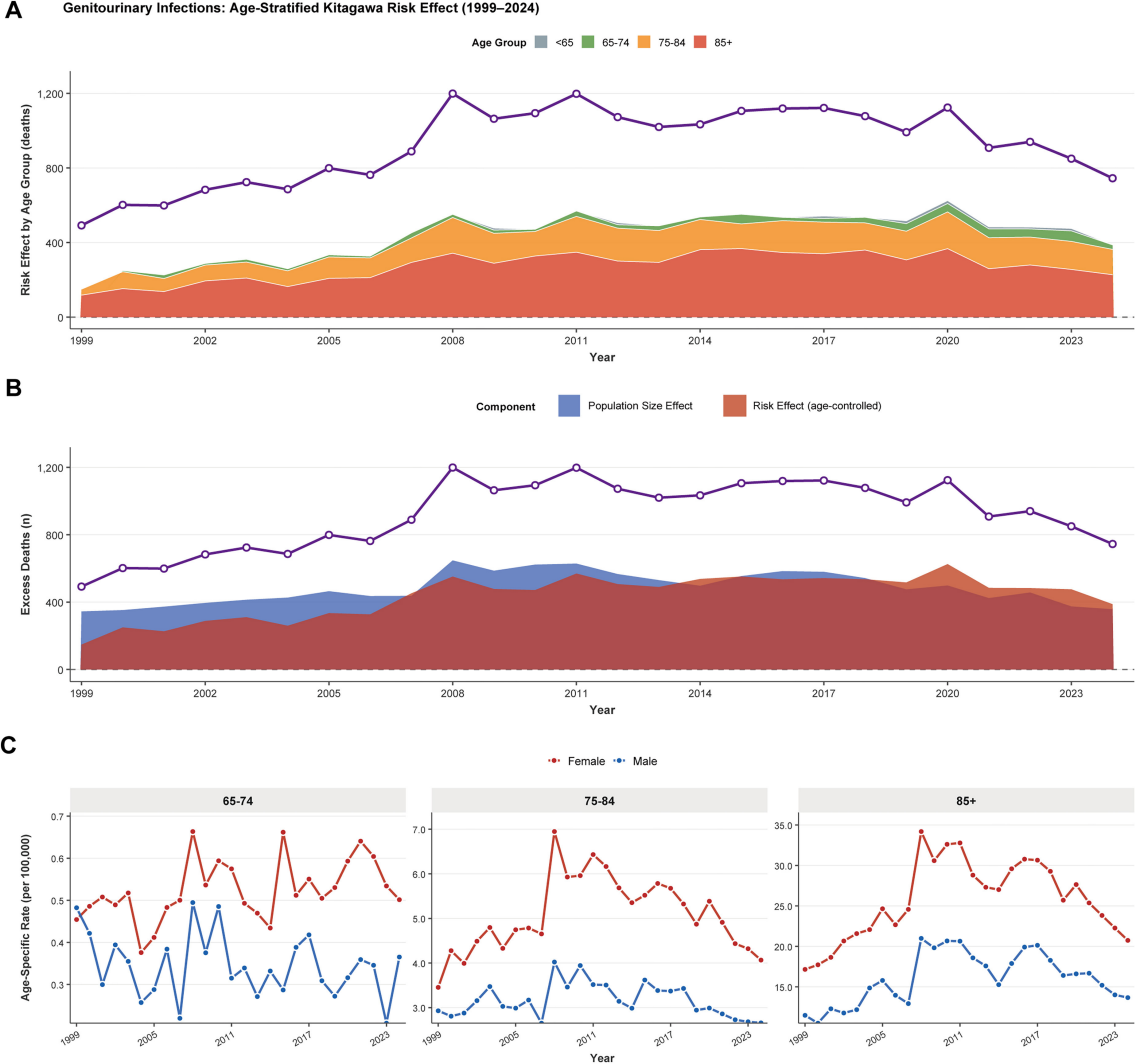
Age-stratified kitagawa decomposition of genitourinary infection-associated Alzheimer’s disease mortality, United States, 1999–2024. **(A)** Age-stratified Kitagawa risk effect for genitourinary infection-associated AD deaths, stratified by age group ( < 65, 65–74, 75–84, ≥ 85 years). **(B)** Annual Kitagawa decomposition of the female excess death burden in genitourinary infections into population size effect and age-controlled risk effect **(C)** Age-specific mortality rates by sex across three elderly age strata (65–74, 75–84, and ≥ 85 years). AD, Alzheimer’s disease; CI, confidence interval.

Genitourinary infections stood apart as the only subtype in which a substantial share of the female excess was attributable to higher age-specific female rates rather than to population age structure (Figures 5A,B). The cumulative risk effect was strongly positive (+11,337; 47.4%), nearly matching the population size effect (+12,566; 52.6%)—a near-equal split unique among all subtypes—and showed a monotonically increasing gradient with age ( ≥ 85 years: +7,066; 75–84 years: +3,556; 65–74 years: +615; < 65 years: +101). The OLS slope of this risk component over calendar time was positive (+12.16 deaths/year); this increase was significant under HAC-robust inference (*p* = 0.004) but not under the AR(1)-GLS specification (*p* = 0.483), in which the autoregressive parameter approached the non-stationary boundary (φ≈ 1; Durbin–Watson 0.59), and it is therefore best characterized as directionally consistent but of model-dependent significance. The substantial positive cumulative risk effect and its age gradient—the features distinguishing genitourinary infection from all other subtypes—are descriptive decomposition quantities and do not depend on this trend inference. The mechanism underlying the higher female age-specific rate cannot be identified from death-certificate data (see Discussion). Age-specific genitourinary mortality rates by sex across the three elderly strata are shown in Figure 5C. Septicemia/sepsis occupied an intermediate position, with the population size effect dominating (89.5%; +11,381) and a modest inconsistent risk effect across age strata that did not reach statistical significance (Supplementary Figure S6).

### Racial/ethnic disparities in infection-associated Alzheimer’s disease mortality

**FIGURE 6:**
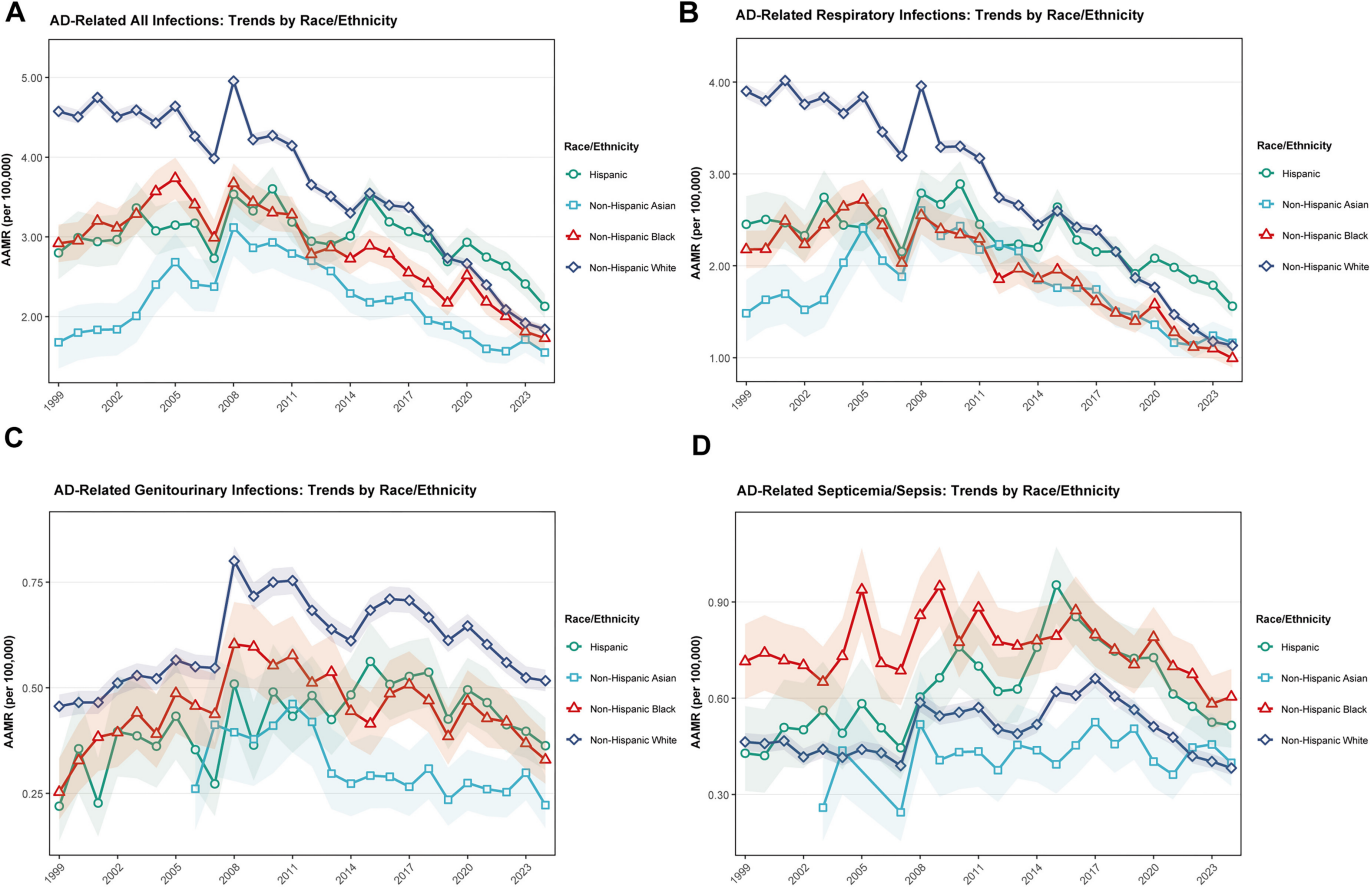
Racial/ethnic disparities in infection-associated Alzheimer’s disease mortality, United States, 1999–2024. Age-adjusted mortality rates (AAMR, per 100,000 population) for infection-associated Alzheimer’s disease deaths by race/ethnicity across four major infection subtypes: **(A)** all infection-associated AD deaths combined, **(B)** respiratory system infections, **(C)** genitourinary infections, and **(D)** septicemia/sepsis. AAMR, age-adjusted mortality rate; AD, Alzheimer’s disease; AAPC, average annual percent change; CI, confidence interval.

Racial/ethnic disparities showed heterogeneous trajectories across infection subtypes (Figure 6 and Supplementary Table S10). For all infections combined, the racial hierarchy inverted over the study period: Non-Hispanic White patients, who had the highest AAMR in 1999 (4.574 per 100,000), achieved the steepest decline (−59.7%; 1.842 per 100,000 in 2024), while Hispanic patients recorded the highest AAMR by 2024 (2.129 per 100,000) despite a more modest decline (−23.9%; Figure 6A). In absolute terms, Hispanic and Non-Hispanic Asian/Pacific Islander deaths increased by +184.8 and +336.2%, respectively—largely reflecting rapid elderly population growth rather than worsening age-specific risk (Supplementary Table S12). Respiratory infections followed a similar convergent pattern, with Hispanic patients recording the smallest decline (−36.3%) and highest AAMR by 2024 (1.561 per 100,000; Figure 6B). Genitourinary infections showed the widest racial disparity: Non-Hispanic White patients maintained the highest AAMR throughout (+13.3%), while Hispanic and Non-Hispanic Black patients showed the largest proportional increases (+65.2 and +30.2%, respectively), and absolute deaths rose across all racial groups (Figure 6C). For septicemia/sepsis, Non-Hispanic Black patients carried the highest AAMR throughout the entire study period (0.715 → 0.604 per 100,000)—a pattern distinct from all other subtypes—while Non-Hispanic Asian/Pacific Islander and Hispanic AAMRs rose (+53.9 and +20.4%; Figure 6D).

A formal between-group test indicated that this ranking reflected a statistically significant difference in age-adjusted rate rather than point-estimate separation alone. In 2024, the Hispanic all-infection AAMR (2.129 per 100,000) significantly exceeded that of Non-Hispanic White patients (difference +0.287, 95% CI 0.132–0.443; *z* = 3.63; *p* = 2.8 × 10^−4^) and, indeed, that of every other group (vs. Non-Hispanic Black, *p* = 9.5 × 10^−5^; vs. Non-Hispanic Asian/Pacific Islander, *p* = 1.0 × 10^−7^; Supplementary Table S11), with the Hispanic and Non-Hispanic White trajectories crossing in 2020. Because the AAMR is age-standardized, this rate difference is independent of the population growth driving the parallel increase in absolute Hispanic deaths (Supplementary Table S12); the inversion reflects a slower decline in the Hispanic rate (–23.9%) than in the Non-Hispanic White rate (–59.7%) rather than any rise in Hispanic age-specific risk. Subtype-specific tests (Supplementary Table S11) indicated a significantly higher Hispanic respiratory AAMR (*p* = 2.2 × 10^−5^) and the significantly higher Non-Hispanic White genitourinary AAMR (*p* = 6.1 × 10^−6^); for septicemia/sepsis, Non-Hispanic Black patients retained the highest point estimate but their separation from the second-ranked group was not statistically significant in 2024 (*p* = 0.11). For Non-Hispanic Asian/Pacific Islander patients, the +336.2% rise in absolute infection-associated AD deaths similarly reflects the very rapid growth and aging of this elderly population rather than an increase in age-specific risk, as the corresponding age-adjusted rate did not rise over the study period.

### Urbanization disparities in infection-associated Alzheimer’s disease mortality

**FIGURE 7:**
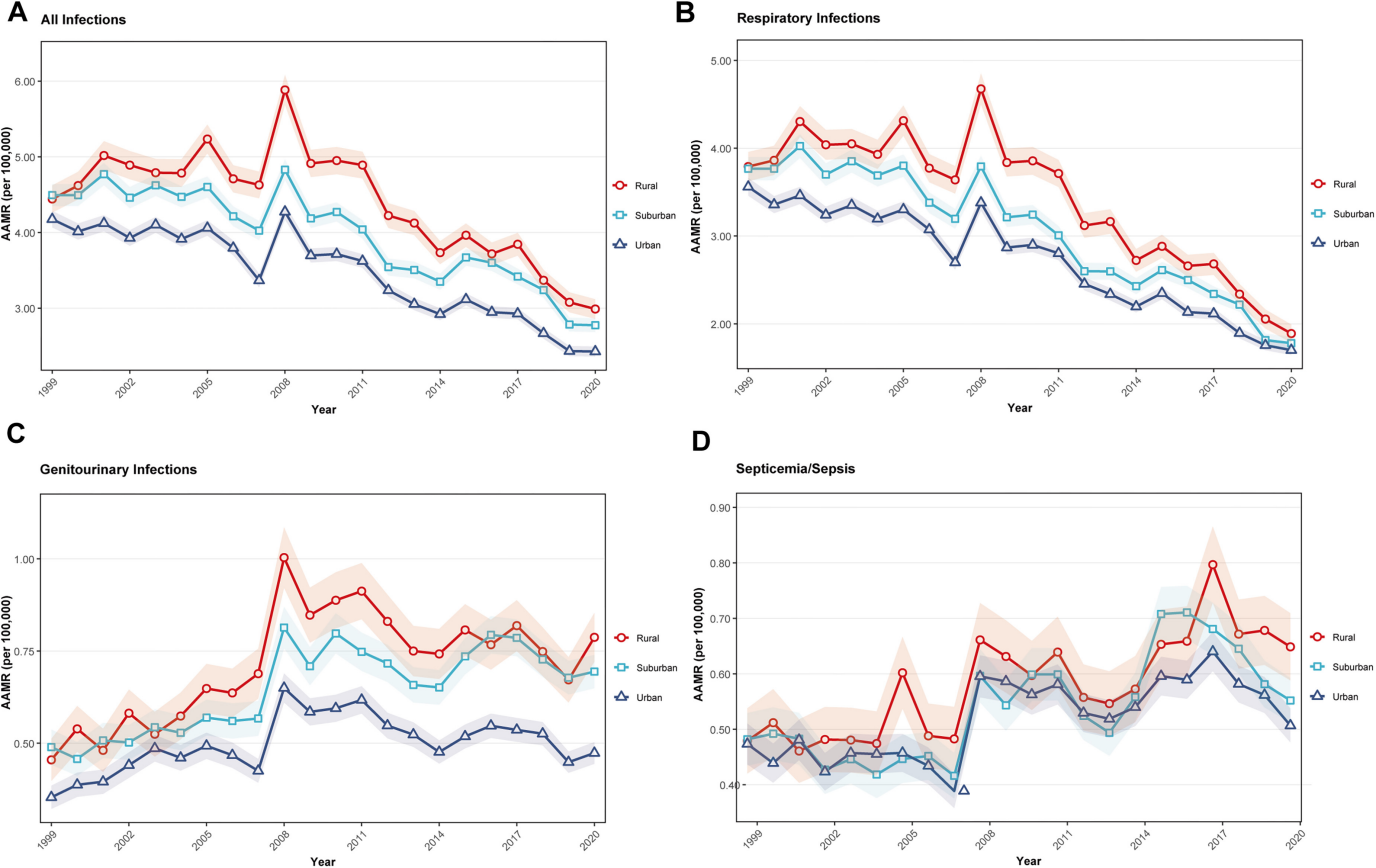
Urbanization-level variation in age-adjusted infection-associated Alzheimer’s disease mortality rates, United States, 1999–2020. Age-adjusted mortality rates (AAMR, per 100,000 population) for infection-associated Alzheimer’s disease deaths across three urbanization levels for four major infection subtypes: **(A)** all infection-associated AD deaths combined, **(B)** respiratory system infections, **(C)** genitourinary infections, and **(D)** septicemia/sepsis. AAMR, age-adjusted mortality rate; AD, Alzheimer’s disease; AAPC, average annual percent change; CI, confidence interval.

Infection-associated AD mortality declined across all three urbanization levels, with urban areas recording the steepest reduction (−41.9%; AAPC −2.45%) and rural areas the most modest (−32.7%; AAPC −1.80%), resulting in a widening rural–urban disparity over time (Figure 7A, Supplementary Figure S7 and Supplementary Table S13). Respiratory infections showed the most geographically uniform pattern, with all three urbanization strata achieving near-identical declines of 50–53% (AAPC range: 0.30 percentage points), suggesting broadly equitable access to respiratory infection prevention across residential settings (Figure 7B). The most pronounced urbanization gradient was observed for genitourinary infections: rural areas recorded the steepest increase (0.455 → 0.787 per 100,000; +73.1%; AAPC +2.81%), followed by suburban (+41.9%) and urban areas (+34.0%), with all three AAPCs statistically significant and absolute deaths rising across all strata (Figure 7C). For septicemia/sepsis, rural areas were the only group with a statistically significant increasing AAPC (+1.93%; 95% CI +1.28 to +2.59), while suburban and urban AAPCs were positive but non-significant (Figure 7D).

### Geographic variation in infection-associated Alzheimer’s disease mortality

**FIGURE 8:**
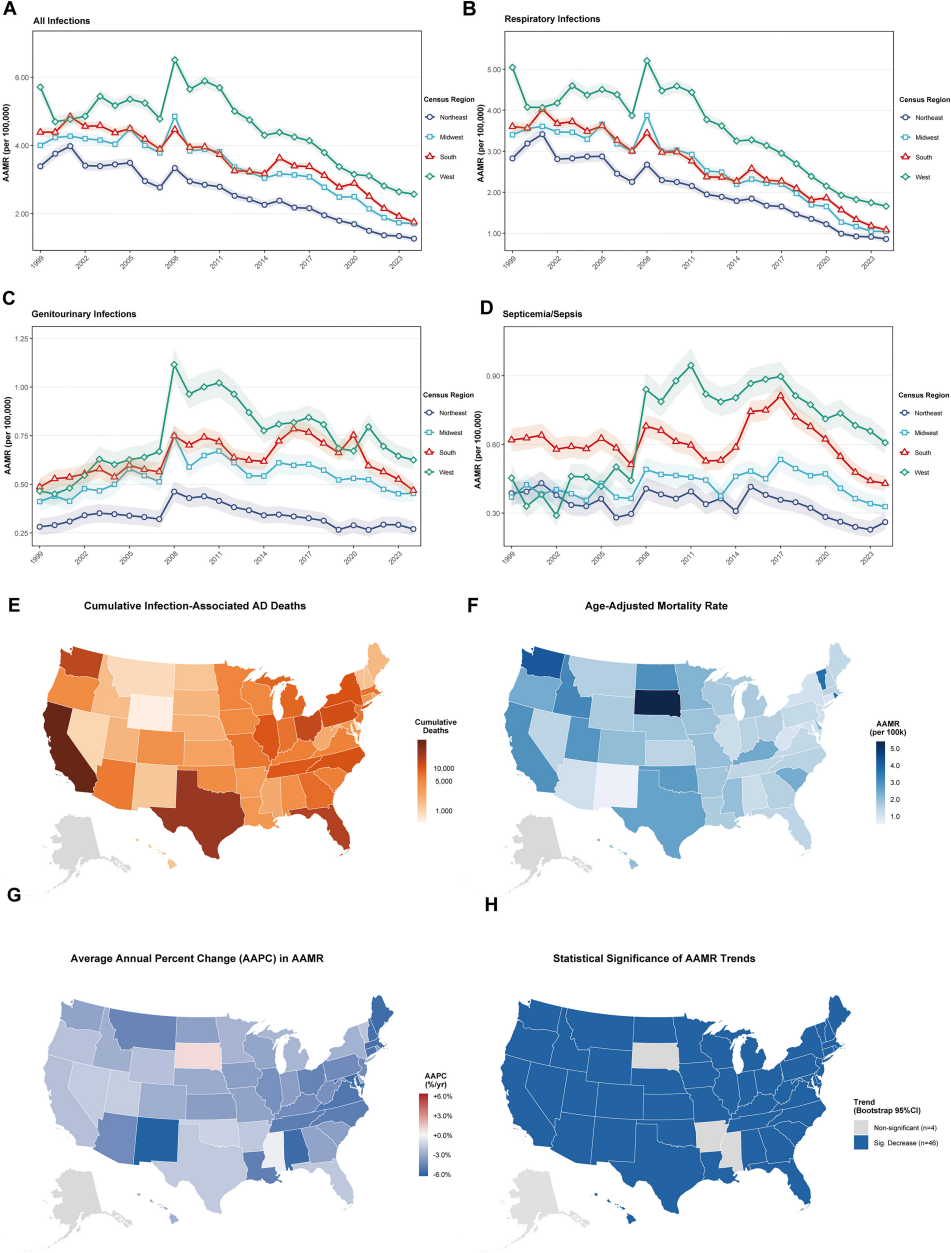
Geographic variation in infection-associated Alzheimer’s disease mortality, United States, 1999–2024. **(A–D)** Age-adjusted mortality rates (AAMR, per 100,000 population) by US Census region for four infection subtypes: **(A)** all infections combined, **(B)** respiratory infections, **(C)** genitourinary infections, and **(D)** septicemia/sepsis; shaded bands represent 95% confidence intervals. **(E)** Choropleth map of cumulative infection-associated AD deaths by state, 1999–2024. **(F)** State-level AAMR in the most recent available year. **(G,H)** State-level average annual percent change (AAPC) in AAMR, estimated by joinpoint regression on log-transformed AAMR with bootstrap 95% confidence intervals. AAMR, age-adjusted mortality rate; AD, Alzheimer’s disease; AAPC, average annual percent change; CI, confidence interval.

All four Census regions recorded significant declines in infection-associated AD AAMR, with the Northeast declining most steeply (AAPC −4.11%) and the West most modestly (AAPC −1.53%; Figure 8A and Supplementary Table S14). Notably, age-adjusted risk and absolute death counts diverged: the South consistently recorded the largest annual death counts, while the West carried the highest AAMR throughout—a pattern persistent across all four major subtypes (Supplementary Figure S8). Respiratory infections showed the most geographically uniform decline, with all four regions achieving reductions of 67–70% and comparable AAPCs (Figure 8B). Regional divergence was most pronounced for genitourinary infections: the West was the only region with a significantly increasing AAPC (+3.20%; 95% CI +0.69 to +5.72), with annual deaths more than doubling (245 → 600), while the remaining regions showed near-flat non-significant trajectories (Figure 8C). For septicemia/sepsis, the West again diverged, with AAMR rising from 0.453 to 0.607 per 100,000 and annual deaths increasing sharply (+140.9%), while the Northeast was the only region with a statistically significant decline (AAPC −1.90%; 95% CI −3.03 to −0.77; Figure 8D).

State-level analysis revealed substantial heterogeneity beyond regional patterns (Figures 8E,H and Supplementary Table S15). California, Texas, and Florida accounted for the largest cumulative death burdens, reflecting population size rather than disease risk (Figure 8E). The highest age-adjusted rates were concentrated in upper Midwest and Western states, with South Dakota recording the highest AAMR in the most recent year (5.38 per 100,000), while the lowest rates were observed in New Mexico (0.54), Connecticut (0.96), and New York (1.06; Figure 8F). Overall, 46 of 50 states achieved statistically significant declining AAPCs, with the fastest improvements in New Mexico (AAPC −6.27%), Maine and Alabama (both −5.76%), and Maryland (−5.74%; Figures 8G,H). South Dakota was the only state with a positive AAPC (+0.85%).

### Projected burden of infection-associated AD mortality, 2025–2034

BSTS local linear trend models were selected as the primary projection method on the basis of superior prediction interval calibration (near-nominal 95% PI coverage, to which wide intervals contributed) over ARIMA and log-linear extrapolation (50–75% coverage) in holdout validation (training: 1999–2018; validation: 2019–2024; Supplementary Figures S9, S10; Supplementary Table S16). All three models exhibited systematic positive forecast bias during the holdout period, with observed deaths consistently falling below projected levels; this bias varied across subtypes and constitutes a limitation of out-of-sample point prediction even if driven in part by pandemic-era structural changes. Sensitivity analyses under optimistic and pessimistic scenarios are presented in Supplementary Figure S11.

**FIGURE 9:**
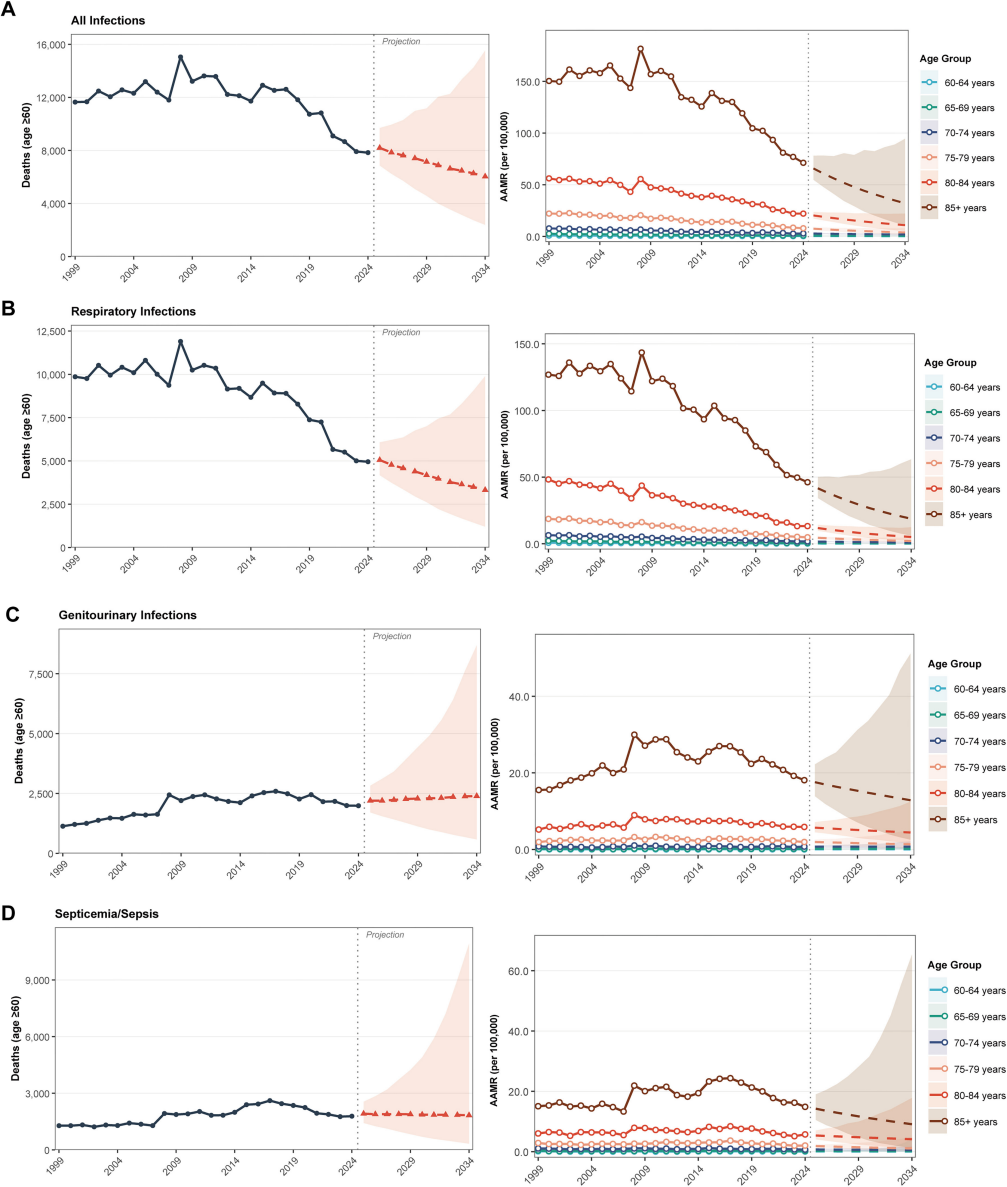
Projected infection-associated Alzheimer’s disease mortality, United States, 2025–2034. Each panel shows projections for one infection subtype: **(A)** all infection-associated AD deaths combined, **(B)** respiratory infections, **(C)** genitourinary infections, and **(D)** septicemia/sepsis. Projections were generated using Bayesian Structural Time Series (BSTS) local linear trend models fitted to log-transformed age-specific mortality rates, with projected deaths calculated by applying predicted rates to US Census Bureau national population projections (2014–2060). AD, Alzheimer’s disease; BSTS, Bayesian Structural Time Series; PI, prediction interval.

For all infection-associated AD deaths combined, projected annual deaths were estimated to peak at 8,195 in 2025 (95% PI: 6,863–9,695) before declining to 6,039 by 2034 (−26.3%), yielding a cumulative burden of 70,541 deaths (95% PI: 44,326–119,538) over 2025–2034 (Figure 9A). The transient near-term increase reflects rapid ≥ 85-year population growth driven by aging baby-boomer cohorts, which sustains absolute death counts even as age-specific rates continue to fall. Respiratory infections were projected to decline most steeply (5,053 → 3,324 deaths; −34.2%; cumulative 41,209; Figure 9B). Genitourinary infections were the only subtype with a projected increase over the forecast horizon (2,202 → 2,392 deaths; +8.6%; cumulative 22,926; Figure 9C); this directional signal was consistent across all three scenario assumptions (Supplementary Figure S11), although the projection is most informative over the near-term horizon (2025–2027), beyond which prediction-interval width increases substantially. Septicemia/sepsis was projected to remain near-stable (1,919 → 1,834 deaths; −4.4%; cumulative 18,759; Figure 9D). Across all subtypes, the ≥ 85-year group dominated projected mortality, with mean projected rates of 47.16 per 100,000 substantially exceeding all younger strata. Prediction intervals reflect rate uncertainty only; true uncertainty incorporating population projection uncertainty is wider than reported. Prediction-interval width increased steeply with forecast horizon for all subtypes (relative width 0.35–0.59 in 2025 rising to 2.18–5.77 in 2034; Supplementary Table S17), exceeding the point estimate itself by approximately 2030. The septicemia/sepsis projection was the widest (relative width 5.77 in 2034; upper bound ≈ 6-fold the point estimate) and should be interpreted as providing no evidence of a clear directional trend rather than as a precise forecast; it is materially less informative than the genitourinary projection. The projections should accordingly be read as exploratory trend-continuation scenarios, most informative in the near term (2025–2027) and best interpreted in terms of direction and relative magnitude rather than as precise long-range forecasts. The near-term directional signal for genitourinary infections, in particular, is supported not by a narrow interval but by its consistency across all three scenario assumptions (Supplementary Figure S11), by its demographic driver (growth of the ≥ 85-year population applied to a non-declining rate), and by its concordance with the upstream epidemiological trends documented elsewhere in this study.

### Sensitivity analyses results

#### Robustness to cause-of-death coding position

Under the reciprocal N-definition—in which infection constituted the underlying cause and AD appeared in any contributing field—the six infection subtypes were mutually exclusive and proportions summed to 100% in both 1999 (*N* = 6,265; Respiratory 71.3%, Genitourinary 14.7%, Septicemia/Sepsis 9.9%, Digestive 2.5%, Others 1.1%, Opportunistic 0.5%) and 2024 (*N* ≈ 1,095; Respiratory 48.1%, Septicemia/Sepsis 23.8%, Digestive 17.1%, Others 8.7%, Opportunistic 1.8%, Genitourinary < 1%), confirming that the > 100% proportions in Figures 1C,D are an inherent feature of the multiple-cause-of-death framework, not an analytical error (Supplementary Table S6).

The N-definition proved structurally unsuitable as a longitudinal comparator for two reasons (Supplementary Figure S12A). First, abrupt single-year discontinuities in 2008 were confined to the respiratory and genitourinary subtypes: respiratory AAMR fell 75.2% in a single year (2007: 1.020 per 100,000; 2008: 0.253; deaths −74.4%), and genitourinary deaths collapsed from 804 to 12 (−98.5%) over the same interval, the genitourinary series being reportable only for 1999–2007 and suppressed or unreliable in all 17 subsequent years; septicemia/sepsis showed no comparable collapse. Both breaks coincide with documented 2008 changes to NCHS ICD-10/ACME coding conventions: a revised classification of pneumonia co-documented with chronic lower respiratory disease (Miniño et al., 2011), and an ACME decision-table update reassigning urinary tract infection away from underlying-cause status when co-documented with prostate cancer or cerebrovascular disease under Selection Rule 3 (National Center for Health Statistics, 2008). Both changes act on underlying-cause selection alone and are therefore invisible to the any-mention P-definition. Second, and separately from these two abrupt breaks, the share of infection-associated AD deaths captured under the N-definition declined gradually across all six subtypes over the full study period (the annual N/P death ratio fell significantly in all six; e.g., respiratory −0.017 and septicemia −0.015 per year, both *p* < 0.001), consistent with the secular rise in AD as a selected underlying cause of death noted in the Introduction rather than with a single dateable rule change. The any-mention P-definition is unaffected by the specific underlying-cause reassignment mechanisms identified above, although it remains potentially susceptible to broader temporal changes in co-documentation practices that cannot be evaluated using aggregate CDC WONDER data (Supplementary Figure S12B).

Where the two definitions could be more directly compared—the post-2008 window 2009–2024—the interpretable subtypes were directionally concordant. Respiratory, septicemia/sepsis, and digestive infections all declined under both definitions (respiratory N AAPC −4.30% vs. P −6.55%; septicemia N −3.61% vs. P −1.73%; digestive N −6.21% vs. P −4.24%; sign agreement in all three, with overlapping 95% CIs for respiratory and septicemia), and their annual AAMR trajectories were positively rank-correlated (Spearman ρ = 0.79, 0.65, and 0.86, respectively). Digestive infection displayed the same biphasic shape under both definitions (rising to a peak around 2009, then declining), reinforcing this concordance. The two lowest-count subtypes (opportunistic, with 12 of 26 years unreliable, and “other” infections) were too sparse or noisy under the N-definition for a stable comparison. Genitourinary infection could not be evaluated under the N-definition at all: its underlying-cause series was reportable only for 1999–2007 (≈830 deaths/year; 919 in 1999) and disappeared thereafter for the documented coding reason identified above. Its rising burden is therefore established solely under the P-definition, which—because it captures infections regardless of whether they appear as underlying or contributing causes of death—is the most appropriate framework available for evaluating this subtype, and which continued to capture 1,200–2,400 genitourinary deaths per year throughout 2008–2024 (Supplementary Figure S12C).

### Scope and impact of suppressed-count imputation

Across the 1,190 stratum-by-subtype-by-year cells comprising the race/ethnicity (1999–2024; 26 years per cell) and urbanization (1999–2020; 22 years per cell) analyses, 169 (14.2%) were suppressed by CDC WONDER [race/ethnicity, 154/728 (21.2%); urbanization, 15/462 (3.2%); Supplementary Table S3]. Suppression was concentrated in the smallest strata (Non-Hispanic Asian/Pacific Islander, 36.8%; Hispanic, 26.9%; Non-Hispanic Black, 20.9%; rural, 8.4%; urban and suburban, 0.6% each) and almost exclusively in the three lowest-incidence subtypes (opportunistic, digestive, and other infections), which together accounted for 166 of the 169 suppressed cells (98.2%); only three suppressed cells fell in a core subtype, all three within the Non-Hispanic Asian/Pacific Islander series (genitourinary, 2/26; septicemia/sepsis, 1/26). The subtypes underpinning the principal findings contained no or near-zero suppression: the rural genitourinary series—the headline urbanization finding—together with the rural all-infection, rural septicemia, and Non-Hispanic Asian/Pacific Islander all-infection and respiratory series each contained no suppressed cells (0/22 or 0/26), while the Non-Hispanic Asian/Pacific Islander septicemia and genitourinary series contained one and two suppressed cells, respectively (Supplementary Table S3). Re-estimation of the crude-rate AAPC under suppressed-count assumptions of 1, 5, and 9 and under complete-case analysis yielded an identical trend direction for every subgroup, with significance unchanged except in the single small-count Non-Hispanic Asian/Pacific Islander genitourinary series (Supplementary Figure S13).

### Autocorrelation-robust inference

The Durbin–Watson statistic confirmed positive residual autocorrelation in most component series. Under HAC-robust standard errors the principal decomposition trends remained significant, including the all-infection and respiratory risk-effect trends (both *p* < 0.001). For the genitourinary risk-effect trend, however, the two specifications diverged: the OLS slope (+12.16 deaths/year) was significant under HAC (*p* = 0.004) but not under AR(1)-GLS (*p* = 0.483), in which the autoregressive parameter reached its boundary (φ≈ 1). Because a boundary AR(1)-GLS fit approximates first-differencing and, at 26 annual observations, cannot be reliably distinguished from a trend-stationary series with strong but stationary autocorrelation, we treat neither specification as authoritative for this series and describe its temporal trend as of model-dependent significance rather than as established; the substantial positive cumulative genitourinary risk effect (+11,337; 47.4%) and its monotonic age gradient are descriptive decomposition quantities and are unaffected by this divergence. Separately, the slope of the annual female-minus-male genitourinary death-count difference was not significant under HAC (*p* = 0.072) and is not interpreted as a robustly widening annual difference (Supplementary Table S7).

### Complementary mixed-effects count model

In a complementary mixed-effects negative binomial model of death counts with population offsets, all three interactions of interest were significant: calendar year × infection subtype (χ^2^(3) = 864.2, *p* < 0.001), calendar year × sex (χ^2^(1) = 9.8, *p* = 0.002), and infection subtype × sex (χ^2^(3) = 73.3, *p* < 0.001). Model-based annual incidence-rate ratios reproduced the joinpoint trends (respiratory –4.83% per year; genitourinary +0.46% per year, the only rising series; all infections –3.60% per year), and the infection subtype × sex interaction localized the female excess to genitourinary infection (female-to-male rate ratio 1.58; *p* < 0.001). Estimates were unchanged under a crossed calendar-year random effect (Supplementary Table S18).

## Discussion

In this nationwide population-based analysis spanning more than 2.3 million AD-related deaths over 26 years, we observed a striking divergence between overall AD mortality and infection-associated AD mortality in the United States. While overall AD mortality continued to rise substantially, infection-associated mortality declined by more than half at the population level. However, this overall improvement concealed marked heterogeneity across infection subtypes. Respiratory infection mortality declined steeply and consistently across demographic and geographic strata, whereas genitourinary infections emerged as the only major subtype with a persistently increasing burden in both absolute deaths and age-adjusted mortality risk. This increase disproportionately affected women and Hispanic individuals (1999–2024) and rural populations (1999–2020), and was the only subtype for which trend-continuation projections suggested a further increase, particularly over the near-term horizon, despite ongoing declines in other infection categories. Together, these findings suggest that the epidemiology of infection-associated mortality in AD is undergoing an important transition, in which traditional respiratory complications are gradually receding while genitourinary infections are emerging as an increasingly important and potentially preventable contributor to late-stage disease burden.

The relationship between dementia and infection is likely bidirectional. Neurodegeneration substantially increases susceptibility to severe infection through frailty, dysphagia, immobility, malnutrition, and dependence on institutional care (Kalia, 2003). In parallel, systemic infection and peripheral inflammation may further exacerbate neuroinflammatory and neurodegenerative processes, creating a potentially self-reinforcing cycle between dementia progression and infection vulnerability (McManus and Heneka, 2017). Previous epidemiological studies in AD have largely focused on pneumonia-related mortality and consistently reported declining respiratory mortality over recent decades (Bouza et al., 2019; Faheem et al., 2026; Janbek et al., 2021). The present study extends this framework by systematically examining six major infection subtypes across the entire 1999–2024 period, incorporating demographic disparities, geographic variation, COVID-era effects, and future projections within a unified multiple-cause-of-death framework. This broader perspective revealed that infection-associated mortality trends in AD are no longer dominated by a single respiratory narrative, but instead reflect increasingly divergent trajectories across infection categories.

The decline in respiratory infection-associated AD mortality represented the most consistent and geographically equitable improvement identified in this study. Respiratory AAMRs fell by more than two-thirds over the study period, with comparable declines observed across all Census regions and urbanization strata. Several converging factors likely contributed to this sustained reduction, including increased uptake of influenza and pneumococcal vaccination, improvements in dysphagia recognition and aspiration prevention, and the progressive institutionalization of infection-control practices within long-term care facilities (Alagiakrishnan et al., 2013; Muder, 1998; Stevenson et al., 2001). The COVID-19 era appeared to accelerate these trends further, likely through the combined effects of non-pharmaceutical interventions, suppression of circulating respiratory pathogens, and enhanced infection prevention practices in nursing homes and hospitals (Lai et al., 2021; Olsen et al., 2021). Nevertheless, respiratory infections remained the single largest contributor to infection-associated AD mortality throughout the study period and are projected to account for more than 40,000 deaths over the coming decade, underscoring the continuing importance of respiratory infection prevention in this highly vulnerable population.

In contrast to the sustained improvement observed for respiratory infections, genitourinary infections emerged as the most concerning and clinically actionable finding of this analysis. Genitourinary mortality increased in both absolute and relative terms, widened across sex (through 2024) and rural–urban strata (through 2020), and was the only infection subtype for which trend-continuation projections suggested a further increase, particularly over the near-term horizon. This trajectory is biologically coherent: dementia amplifies urinary tract infection risk through urinary incontinence, impaired toileting ability, prolonged catheter dependence, and—in women—anatomical vulnerability and vaginal atrophy, with UTIs occurring up to 30 times more frequently in women than men and carrying substantially elevated mortality risk in cognitively impaired patients (Kim et al., 2026; Lai et al., 2024). Importantly, the Kitagawa decomposition indicated that the female excess in genitourinary mortality was not attributable to population age structure alone: a substantial share reflected higher age-specific female rates (cumulative risk effect 47.4% of the female excess), distinguishing this subtype from all others, in which the apparent female excess was attributable primarily to the greater number of older women in the at-risk population rather than to higher age-specific rates. The direction of change in this risk component over time was consistent across analyses, but its statistical significance was sensitive to the treatment of serial autocorrelation, and we therefore do not interpret the temporal slope as firmly established. As an arithmetic decomposition, moreover, this analysis apportions the death-count difference between rate and structure components and does not identify a biological, clinical, or individual-level mechanism, nor exclude between-sex differences in catheter exposure, institutionalization, healthcare use, diagnosis, or certification practices.

The structural dimensions of this burden are equally concerning. Despite national AHRQ-funded initiatives to implement evidence-based CAUTI prevention bundles in nursing homes—encompassing prompt catheter removal, catheter alternatives, and antimicrobial stewardship—up to 70% of catheter-associated UTIs remain preventable under adherence to recommended guidelines, pointing to a persistent implementation gap rather than an absence of effective strategies (Mody et al., 2015). This gap was most acutely exposed during the COVID-19 pandemic: whereas respiratory and septicemia infections fell well below pre-pandemic projections, genitourinary infections showed by far the smallest cumulative deficit and were the only subtype whose 2020 deaths did not fall below projection (a deviation within the 95% prediction interval, not a significant excess). This relative attenuation may reflect several non-exclusive factors, including the nursing home staffing shortages and visitor restrictions that compromised the hygiene-dependent basic care on which urinary infection prevention depends (McGarry et al., 2020; McGilton et al., 2020), and an anatomically driven etiology less susceptible to respiratory-focused non-pharmaceutical interventions. The widening rural–urban disparity in genitourinary mortality further reflects a structural care deficit consistent with documented deficiencies in rural long-term care infection control resources and geriatric specialist access (Arsenault-Lapierre et al., 2023; Gulline et al., 2025). As the only infection subtype for which projections suggest a continued near-term increase in absolute mortality, genitourinary infections may represent an important and potentially addressable component of AD infection prevention strategy—an inference grounded in the external prevention literature rather than demonstrated by the present data.

Several important demographic disparities merit attention. Racial and ethnic patterns underwent a striking inversion over the study period, with Non-Hispanic White patients — who carried the highest AAMR in 1999 — achieving the steepest declines, while Hispanic patients recorded the highest observed age-adjusted mortality rate by 2024. This shift likely reflects the intersection of rapid Hispanic elderly population growth with persistent structural disadvantages in healthcare access and insurance coverage, compounding what prior literature describes as an attenuation of the Hispanic mortality paradox at older ages in the context of complex terminal illness (Markides and Eschbach, 2005; Mehta and Yeo, 2017; Palloni and Arias, 2004). Non-Hispanic Black patients carried persistently elevated septicemia mortality throughout the study period — a pattern consistent with well-documented structural inequities in sepsis care driven by lower vaccination coverage, differential access to evidence-based treatment, and systemic disparities at community and facility levels (DiMeglio et al., 2018; Dombrovskiy et al., 2007). The divergence between absolute death counts and age-adjusted rates across minority groups — with Hispanic and Asian/Pacific Islander deaths rising substantially despite stable or declining per-capita risk — has direct policy implications: resource-allocation decisions based solely on age-adjusted rates risk systematically underprioritizing communities where the absolute burden is expanding most rapidly. This ranking inversion was supported by a formal between-group test: by 2024 the Hispanic age-adjusted rate significantly exceeded that of all other groups. Because age-adjusted rates remove differences in population size and age structure, this inversion is a property of age-specific risk and is therefore independent of, and complementary to, the population-growth-driven increase in absolute Hispanic deaths; it arose because the Hispanic rate declined more slowly than the Non-Hispanic White rate, not because Hispanic risk rose.

Several strengths of this study merit acknowledgment. The analysis draws upon a nationally representative dataset spanning 26 years and more than 300,000 infection-associated AD deaths, providing sufficient statistical power to characterize long-term trends across multiple demographic and geographic strata simultaneously. The integration of multiple-cause-of-death analysis, Kitagawa decomposition, and Bayesian structural time series forecasting enabled simultaneous evaluation of historical trends, sex-specific risk attribution, and future burden projections across infection subtypes. In addition, the inclusion of the complete COVID-19 period through 2024 provides a uniquely comprehensive assessment of pandemic-era effects on infection-associated AD mortality. Limitations include reliance on ICD-10 death certificate data subject to coding variability — particularly for septicemia following the 2016 Sepsis-3 definition revision — and the absence of individual-level data on nursing home residency, AD severity, vaccination history, catheter use, and antibiotic exposure. A further structural limitation of the CDC WONDER platform is that it provides aggregated mortality counts rather than individual-level death records; it is therefore not possible to directly quantify the frequency of co-occurring infection subtypes on individual death certificates, nor to formally test whether temporal shifts in co-documentation practices contributed to the observed changes in subtype composition. Although the any-mention multiple-cause-of-death approach is the standard methodology for this type of population surveillance, and the reciprocal N-definition sensitivity analysis supports the directional consistency of the respiratory, septicemia/sepsis, and digestive trends specifically, this comparison could not be extended to the genitourinary trend itself, which is not assessable under the N-definition after 2008; the possibility that evolving co-documentation practices contributed in part to the changing subtype proportions, including the genitourinary trend, cannot be entirely excluded.

Pathogen-specific findings are descriptive only, given coding availability in fewer than 3% of cases. Projection uncertainty is wider than reported prediction intervals suggest, as population forecast uncertainty is not incorporated. Geographic findings are ecological and should not be interpreted as individual-level causal relationships. A further consideration concerns temporal stability of cause-of-death coding. The sensitivity analysis identified abrupt discontinuities around 2008 in the N-definition data that are absent from the primary P-definition series. Both are attributable to documented NCHS ICD-10/ACME coding-convention changes effective with the 2008 data year: for the respiratory subtype, the reclassification of pneumonia co-documented with a chronic lower respiratory condition away from pneumonia (Miniño et al., 2011); and for the genitourinary subtype, the addition of prostate cancer and cerebrovascular disease to the ACME list of conditions causing inability to control the bladder, which reassigns co-reported urinary tract infections away from underlying-cause status under Selection Rule 3 (National Center for Health Statistics, 2008). Both changes act only on which single condition is selected as the underlying cause and therefore leave the any-mention P-definition unaffected. Although this reinforces the analytical appropriateness of the any-mention framework, the possibility that more gradual shifts in co-documentation practices contributed in part to the observed changes in subtype proportions cannot be entirely excluded.

Death-certificate data are subject to substantial coding variability. AD may be under-recorded or inconsistently designated as an underlying rather than a contributing cause, and infection documentation may vary across certifying institutions, geographic regions, calendar years, and especially during the COVID-19 period, when certification practices shifted markedly. Although the any-mention framework is structurally insensitive to movement of AD or infection between the underlying and contributing positions — the axis of coding change illustrated by the documented 2008 discontinuities described above — variability across institutions, regions, and the pandemic era cannot be fully excluded, and the outcome should therefore be interpreted as documentation-based rather than clinically adjudicated.

Several caveats constrain the 10-year projections. First, during holdout validation all three candidate models exhibited systematic positive forecast bias across 2019–2024, with observed deaths consistently falling below projected levels. Although this overestimation likely reflects in part the pandemic-era mortality deficit documented in our excess-mortality analysis, it nonetheless constitutes a limitation of out-of-sample point prediction: if the post-2019 mortality regime persists — whether through sustained infection-control improvements, competing COVID-19 mortality, or other structural changes — the forward projections may be upwardly biased. Moreover, this bias varied substantially across subtypes (septicemia/sepsis exhibited the largest holdout overestimation), and high prediction-interval coverage (86–100% for BSTS) was achieved in the context of wide intervals, a feature that reflects interval calibration rather than point-prediction accuracy. Second, prediction-interval width grows rapidly with horizon and was validated only to 6 years; beyond 2030 the intervals are unvalidated extrapolations, and for septicemia/sepsis the interval becomes uninformative. The projections should therefore be read as exploratory trend-continuation scenarios under stable epidemiological and coding conditions — most informative in the near term (2025–2027) and best interpreted in terms of direction and relative magnitude — rather than as precise long-range forecasts, and they do not incorporate future structural shocks or population-projection uncertainty. The apparent relative attenuation of the genitourinary deficit during the pandemic should be interpreted cautiously. The 2020 deviation was a modest, non-significant departure that fell within the pre-pandemic 95% prediction interval, and — like the deficits in all other subtypes — does not constitute a statistically significant single-year effect; the relevant evidence is the markedly smaller cumulative genitourinary deficit. Moreover, part of the modest 2020 signal may be attributable to COVID-19–associated urinary-tract-infection documentation: under the any-mention framework, COVID-19 decedents with recorded urinary-tract infection and prolonged catheterisation would be captured as genitourinary infection-associated, a contribution not separable in aggregate data.

Our analysis is based on the co-occurrence of AD and infection-related ICD-10 codes on the same death certificate and therefore cannot establish that the recorded infection caused death, that it was catheter-associated, or that the death was preventable; nor can it capture individual-level determinants such as nursing-home residence, AD severity, catheter exposure, vaccination history, or antimicrobial use. References to catheterisation and CAUTI prevention are drawn from the external clinical literature and are offered as biologically plausible context for the observed mortality patterns rather than as factors measured in the present data. Finally, although 14.2% of cells in the stratified race/ethnicity (1999–2024) and urbanization (1999–2020) analyses were CDC WONDER–suppressed, suppression was concentrated in low-incidence subtypes that do not underpin the principal findings (which derive from series with zero or near-zero suppression), and a sensitivity analysis confirmed that trend directions were robust to alternative treatments of suppressed counts; the age-standardized trends forming the basis of our conclusions are, by construction, independent of the imputed value.

## Conclusion

In conclusion, infection-associated AD mortality has declined substantially over the past quarter century, yet genitourinary infections represent a growing and systematically neglected contributor to late-stage disease burden that is rising in absolute terms, with trend-continuation projections suggesting a possible further increase—particularly over the near-term horizon—and one that, on the basis of external clinical evidence on catheter-associated urinary tract infection prevention, may be substantially preventable. On the basis of the broader clinical literature on infection prevention, these findings point to several actionable priorities: systematic expansion of CAUTI prevention bundles in long-term care facilities, sex-specific continence and bladder health programs for female AD patients, and investment in rural infection prevention infrastructure. Future research should link individual-level clinical data — including catheter use, nursing home residency, and antibiotic stewardship — to infection-specific mortality outcomes in this population. As the baby boom generation enters the highest-risk decade for Alzheimer’s disease, infection-associated deaths in this population represent an urgent and potentially addressable challenge for the public health response to this growing crisis.

## Data Availability

Publicly available datasets were analyzed in this study. This data can be found at: https://wonder.cdc.gov/.

## Footnotes

1
https://wonder.cdc.gov/
