# Supplementary material for: The changing epidemiology of infection-associated mortality in Alzheimer’s disease in the United States, 1999–2024
Source: Front Aging Neurosci. 2026 Jul 30;18:1885030. doi: 10.3389/fnagi.2026.1885030 (PMC13468505; doi:10.3389/fnagi.2026.1885030)
Supplement: Supplementary file 1 [file Data_Sheet_1.zip › Data sheet/supplement Figures.docx]

**Supplementary Material**

**The Changing Epidemiology of Infection-Associated Mortality in Alzheimer’s Disease in the United States, 1999–2024**

**Table of Contents**

**Supplementary Figures**

**Figure S1.** Joinpoint regression analysis of age-adjusted mortality rate trends for AD-related and infection-associated deaths, United States, 1999–2024.

**Figure S2.** Pathogen-level trends in infection-associated Alzheimer’s disease mortality, United States, 1999–2024.

**Figure S3.** Joinpoint regression analysis of age-adjusted mortality rates by pathogen type, United States, 1999–2024.

**Figure S4.** Sex-stratified mortality trends for minor infection subtypes in Alzheimer’s disease, United States, 1999–2024.

**Figure S5.** Age-stratified Kitagawa decomposition of respiratory infection-associated Alzheimer’s disease mortality, United States, 1999–2024.

**Figure S6.** Age-stratified Kitagawa decomposition of septicemia/sepsis-associated Alzheimer’s disease mortality, United States, 1999–2024.

**Figure S7.** Urbanisation-level trends in annual infection-associated Alzheimer’s disease death counts, United States, 1999–2020.

**Figure S8.** Regional trends in annual infection-associated Alzheimer’s disease death counts by US Census region, United States, 1999–2024.

**Figure S9.** Holdout validation of age-specific mortality rate projection models for infection-associated Alzheimer’s disease, ≥85 years age group, United States.

**Figure S10.** Model validation performance: 95% prediction interval coverage and mean absolute percentage error across candidate projection methods.

**Figure S11.** Scenario-based projections of infection-associated Alzheimer’s disease deaths under optimistic, status quo, and pessimistic assumptions, United States, 2025–2034.

**Figure S12.** Sensitivity Analysis: Impact of the 2008 ICD-10 Coding Convention Changes on Infection-Associated Alzheimer's Disease Mortality Under the N-Definition versus P-Definition, United States, 1999–2024.

**Figure S13.** Sensitivity of the crude-rate average annual percent change (AAPC) to assumptions about CDC WONDER–suppressed death counts.

**Figure S1. Joinpoint regression analysis of age-adjusted mortality rate trends for AD-related and infection-associated deaths, United States, 1999–2024.**

(A) Annual percent change (APC) timeline across joinpoint-defined segments for eight categories (AD overall, AD with any infection, and six infection subtypes). Significance levels are annotated within each bar (*p<0.05, **p<0.01, ***p<0.001; ns = not significant). (B) Forest plot of the average annual percent change (AAPC) over the full study period (1999–2024) for all eight categories. Points represent AAPC estimates; horizontal lines denote 95% confidence intervals. Red = significant increase (p<0.05); blue = significant decrease (p<0.05); grey = not significant.AAMR, age-adjusted mortality rate per 100,000; APC, annual percent change; AAPC, average annual percent change; CI, confidence interval; AD, Alzheimer's disease.

**Figure S2. Pathogen-level trends in infection-associated Alzheimer's disease mortality, United States, 1999–2024.**

(A)Annual number of deaths with pathogen-specific coding, stratified by pathogen type (stacked bars). (B) Proportional composition of pathogen-coded deaths by year, expressed as percentage of annual total. (C) Age-adjusted mortality rate (AAMR, per 100,000) for each pathogen type from 1999 to 2024. Pathogen-coded deaths represent approximately 2.4% (7,289 of 308,045 deaths) of all infection-associated AD deaths across the study period. AAMR = age-adjusted mortality rate; CI = confidence interval; G+ = Gram-positive bacteria; G− = Gram-negative bacteria.

**Figure S3. Joinpoint regression analysis of age-adjusted mortality rates by pathogen type, United States, 1999–2024.**

**(A)** Annual percent change (APC) for each joinpoint segment, derived from weighted log-linear regression on log-transformed AAMR. **(B)** Average annual percent change (AAPC) over the full study period for each pathogen type. APC = annual percent change; AAPC = average annual percent change; CI = confidence interval; BIC = Bayesian Information Criterion. *p<0.05; **p<0.01; ***p<0.001.

**Figure S4. Sex-Stratified Mortality Trends for Minor Infection Subtypes in Alzheimer's Disease, United States, 1999–2024.**

(A) digestive system infections, (B) other infections, and (C) opportunistic infections. AAMR, age-adjusted mortality rate per 100,000 population; LOESS, locally estimated scatterplot smoothing; CI, confidence interval.

**Figure S5. Age-Stratified Kitagawa Decomposition of Respiratory Infection-Associated Alzheimer's Disease Mortality, United States, 1999–2024.**

(A) Age-stratified Kitagawa risk effect for respiratory infection-associated AD deaths, stratified by age group (<65, 65–74, 75–84, ≥85 years). (B) Annual Kitagawa decomposition of the female excess death burden in respiratory infections into population size effect and age-controlled risk effect. (C) Age-specific mortality rates by sex (female, red; male, blue) across three elderly age strata (65–74, 75–84, and ≥85 years).AD, Alzheimer's disease; CI, confidence interval.

**Figure S6. Age-Stratified Kitagawa Decomposition of Septicemia/Sepsis-Associated Alzheimer's Disease Mortality, United States, 1999–2024.**

(A) Age-stratified Kitagawa risk effect for septicemia/sepsis-associated AD deaths, stratified by age group (<65, 65–74, 75–84, ≥85 years). (B) Annual Kitagawa decomposition of the female excess death burden in septicemia/sepsis into population size effect (blue) and age-controlled risk effect (orange). (C) Age-specific mortality rates by sex (female, red; male, blue) across three elderly age strata (65–74, 75–84, and ≥85 years). AD, Alzheimer's disease; CI, confidence interval.

**Figure S7. Urbanisation-Level Trends in Annual Infection-Associated Alzheimer's Disease Death Counts, United States, 1999–2020.**

(A) all infection-associated AD deaths combined, (B) respiratory system infections, (C) genitourinary infections, and (D) septicemia/sepsis.

**Figure S8. Regional Trends in Annual Infection-Associated Alzheimer's Disease Death Counts by Census Region, United States, 1999–2024.**

Annual number of infection-associated Alzheimer's disease deaths across four US Census regions for four major infection subtypes: (A) all infection-associated AD deaths combined, (B) respiratory system infections, (C) genitourinary infections, and (D) septicemia/sepsis. AD, Alzheimer's disease.

**Figure S9. Holdout Validation of Age-Specific Mortality Rate Models for Infection-Associated Alzheimer's Disease, ≥85 Years Age Group, United States.**

(A) all infections combined, (B) respiratory infections, (C) genitourinary infections, and (D) septicemia/sepsis. Three methods are compared: BSTS local linear trend (orange), ARIMA (blue), and linear extrapolation on log-transformed rates (green); shaded bands represent 95% prediction intervals. BSTS, Bayesian Structural Time Series; ARIMA, autoregressive integrated moving average; PI, prediction interval.

**Figure S10. Model Validation Performance: 95% Prediction Interval Coverage and Mean Absolute Percentage Error.**

(A) Boxplots of 95% PI coverage across age groups (60–64 through ≥85 years) for three candidate models (BSTS, orange; ARIMA, blue; linear extrapolation, green) across four infection subtypes. (B) Mean Absolute Percentage Error (MAPE) by age group and infection subtype for the 2019–2024 holdout period.BSTS, Bayesian Structural Time Series; MAPE, mean absolute percentage error; PI, prediction interval.

**Figure S11. Scenario Projections of Infection-Associated Alzheimer's Disease Deaths, United States, 2025–2034.**

Projected annual deaths (ages ≥60) under three trend scenarios for four infection subtypes: (A) all infections combined, (B) genitourinary infections, (C) respiratory infections, and (D) septicemia/sepsis. Status quo (red): historical log-rate slope unchanged.

**Figure S12. Sensitivity Analysis**

**(A)** Annual death counts under the N-definition (infection-related ICD-10 code as the underlying cause of death, with Alzheimer's disease [G30] in any multiple-cause field) for six anatomically defined infection subtypes, 1999–2024. **(B)** Overlay of age-adjusted mortality rate (AAMR, per 100,000 population) trends under the P-definition and the N-definition. **(C)** Stacked area chart of absolute annual death counts under the N-definition for all six infection subtypes combined, 1999–2024. AAMR, age-adjusted mortality rate; AD, Alzheimer's disease; ICD-10, International Classification of Diseases, Tenth Revision; NCHS, National Center for Health Statistics; P-definition, primary analysis (AD as underlying cause, infection any-mention); N-definition, sensitivity analysis (infection as underlying cause, AD any-mention); UCD, underlying cause of death.

**Figure S13. Sensitivity of the crude-rate average annual percent change (AAPC) to assumptions about CDC WONDER–suppressed death counts.**
