# Supplementary material for: The changing epidemiology of infection-associated mortality in Alzheimer’s disease in the United States, 1999–2024
Source: Front Aging Neurosci. 2026 Jul 30;18:1885030. doi: 10.3389/fnagi.2026.1885030 (PMC13468505; doi:10.3389/fnagi.2026.1885030)
Supplement: Supplementary file 1 [file Data_Sheet_1.zip › Data sheet/Supplementary Appendix.docx]

**S1. Pathogen-Level Characterisation of Infection-Associated Alzheimer’s Disease Mortality**

Pathogen-specific ICD-10 coding was available for only 7,289 of 308,045 infection-associated Alzheimer’s disease (AD) deaths (2.4%) over the 1999–2024 study period. Because this subset represents a small and potentially non-representative fraction of the total infection-associated burden, the pathogen-level analysis is reported here, in the Supplementary Materials, rather than in the main text, and should be interpreted as descriptive and hypothesis-generating rather than as population-representative estimates.

**S1.1 Supplementary Methods**

Pathogen-coded deaths were classified into four categories based on the ICD-10 codes recorded in the cause-of-death fields: Gram-positive bacteria, Gram-negative bacteria, viral infections, and other pathogens (including fungi, parasites, and unspecified organisms). The full mapping of ICD-10 codes to pathogen categories is provided in Table S2. Given that pathogen-specific coding was available in only 7,289 of 308,045 infection-associated AD deaths (2.4%), pathogen-level findings are presented as descriptive and hypothesis-generating rather than population-representative estimates. Age-adjusted mortality trends for each pathogen category were examined using the same joinpoint regression framework described in the main-text Methods.

**S1.2 Supplementary Results**

Among infection-associated AD deaths, pathogen-specific coding was available in 7,289 cases across the 26-year study period, representing approximately 2.4% of all infection-associated AD deaths (annual range: 147 in 2001 to 462 in 2018). These findings should therefore be interpreted as descriptive and hypothesis-generating rather than representative of the full infective burden in this population (Figure S2, Figure S3).

Despite this limitation, a marked compositional shift was observed between 1999 and 2024 (Figure S2A–S2B). Gram-positive bacteria were the predominant pathogen category in 1999, accounting for 44.7% of pathogen-coded deaths, followed by viral infections (31.6%), Gram-negative bacteria (13.8%), and other pathogens (9.9%). By 2024, this hierarchy had substantially changed: viral infections emerged as the leading category (44.5%), while the proportion attributable to Gram-positive bacteria fell to 23.4% (−21.3 percentage points) and Gram-negative bacteria rose modestly to 20.8% (+7.0 percentage points).

Age-adjusted mortality trends were consistent with these compositional changes (Figure S2C, Figure S3). Viral infections showed the steepest and most sustained increase, with an AAMR rise of +141.9% over the study period (AAPC +4.80% per year; 95% CI +3.24% to +6.38%; p < 0.001). Gram-negative bacteria followed a biphasic pattern: the AAMR rose sharply from 1999 to 2008 (APC +20.49%; p < 0.001), remained broadly stable through 2015, then declined substantially thereafter (APC −12.27% per year; p < 0.001). The overall AAPC for Gram-negative bacteria was +2.53% per year (95% CI +1.39% to +3.67%; p < 0.001). Gram-positive bacteria exhibited a modest but significant decline throughout the period (AAPC −1.36%; 95% CI −2.18% to −0.54%; p = 0.001), while other pathogens showed no meaningful trend (AAPC +0.24%; p = 0.674).
